# Beyond hormone deficiency: a framework for endocrine-immune-niche integration in aging skeletal muscle regeneration

**DOI:** 10.3389/fimmu.2026.1906969

**Published:** 2026-07-29

**Authors:** Laura Perin, Stefano Da Sacco, Yan-Yun Liu, Gregory A Brent, Anna Milanesi

**Affiliations:** 1 Gabriel’s Organization Funding All Renal Research (GOFARR) Laboratory, Children’s Hospital Los Angeles, Division of Urology, Saban Research Institute, Los Angeles, CA, United States; 2 Keck School of Medicine, University of Southern California, Los Angeles, CA, United States; 3 Department of Medicine, Division of Endocrinology, Diabetes, and Metabolism, David Geffen School of Medicine at University of California, Los Angeles–VA Greater Los Angeles Healthcare System, Los Angeles, CA, United States

**Keywords:** aging, endocrine immune network factors, muscle stem cells, skeletal muscle regeneration, thyroid hormone signaling

## Abstract

Thyroid hormone (TH) signaling is widely recognized as an important regulator of skeletal muscle regeneration, yet the mechanisms by which aging alters TH-dependent regenerative responses remain unresolved. Current models generally assume that age-associated regenerative decline reflects reduced thyroid hormone receptor (THR) signaling resulting from alterations in hormone availability, receptor expression, or downstream transcriptional activity. However, several observations challenge this view, including the persistence of THR activity in aged tissues, the limited efficacy of thyroid hormone replacement in restoring regeneration, and the distinct regenerative phenotypes observed in aging and hypothyroidism. Here, we propose the hypothesis that aging does not primarily impair skeletal muscle regeneration through loss of THR signaling, but through disruption of endocrine-immune-niche integration. In this framework, THR signaling functions as a systems-level coordinator that integrates endocrine signals with stromal remodeling, immune responses, and intercellular communication networks required for effective tissue repair. To evaluate whether available evidence is consistent with this hypothesis, we integrate published studies with analyses of publicly available murine single-cell transcriptomic, chromatin-accessibility, and ligand-receptor datasets spanning skeletal muscle regeneration, aging, and hypothyroidism. These associative analyses suggest that substantial components of THR activity remain detectable during aging, whereas coordination between THR signaling, macrophage remodeling, extracellular-matrix programs, inflammatory pathways, and Notch-mediated communication is altered. The findings are associative and do not establish causality; rather, they provide a hypothesis-generating framework for experimental testing. We propose that aging represents a state of impaired endocrine-immune-niche integration in which thyroid hormone signaling remains partially active but becomes increasingly uncoupled from the regenerative programs it normally coordinates. This framework offers a possible unifying explanation for several previously disconnected observations and generates experimentally testable predictions regarding endocrine-immune communication during tissue repair and aging.

## 1 Introduction

Skeletal muscle regeneration declines progressively with aging, yet the mechanisms responsible for this loss of regenerative capacity remain incompletely understood. Among the signaling pathways that regulate regenerative responses, thyroid hormone (TH) signaling has emerged as a critical determinant of muscle repair. Thyroid hormone influences multiple regenerative processes, including muscle stem cell (MuSC) activation, differentiation, metabolic adaptation, tissue remodeling, and immune responses following injury (1–5). During regeneration, TH signaling coordinates cellular responses across myogenic, stromal, and immune populations, facilitating the transition from tissue damage to functional repair (1). The role of TH signaling in regeneration has traditionally been interpreted through a cell-intrinsic framework centered on MuSCs. Within this model, TH regulates regenerative progression through direct transcriptional control of myogenic programs, particularly those governed by MyoD, myogenin, and other myogenic regulatory factors (6, 7). TH receptor α (THRA) directly regulates MyoD transcription and interacts with key components of the myogenic differentiation machinery (7–9), while local control of intracellular T3 availability through the D2/D3 deiodinase system governs the transition between proliferation and differentiation (3, 10, 11). The importance of this pathway is further supported by experimental models demonstrating that hypothyroidism, impaired local thyroid hormone metabolism, or genetic disruption of TH receptors compromise muscle repair and contribute to age-associated declines in muscle function (1, 2, 8, 9, 12). This cell-intrinsic framework has been highly successful in explaining the effects of hypothyroidism and genetic disruption of TH signaling on muscle regeneration.

Aging is associated with extensive remodeling of thyroid hormone biology at multiple levels of the signaling axis, including hormone transport, intracellular metabolism, receptor expression, and TH-responsive gene regulation (13–15). Importantly, these alterations are highly tissue-specific and cell-type dependent. In skeletal muscle, THRA expression declines with age (16), whereas TH transporters and deiodinases may become upregulated (3, 17, 18). At the systemic level, aging is characterized by increased circulating TSH, relative preservation of FT4, and more modest reductions in FT3, suggesting that aging modifies thyroid hormone signaling through complex adaptive mechanisms rather than simple endocrine failure (19).

The prevailing interpretation is that these age-associated alterations in thyroid hormone signaling contribute to regenerative decline through reduced THR activation, diminished transcriptional responsiveness, or defective execution of TH-dependent regenerative programs. However, available evidence does not fully support this prediction. Several components of TH signaling remain preserved in aged skeletal muscle, and studies have reported evidence of local thyroid hormone resistance rather than complete loss of signaling activity (13, 14). Furthermore, thyroid hormone replacement has demonstrated limited ability to restore regenerative competence in aged muscle despite evidence of persistent regenerative impairment (20). Aging and hypothyroidism also produce overlapping but distinct phenotypes (1, 20). Hypothyroidism is characterized by reduced myogenic diversity, cell cycle arrest, and persistent proinflammatory macrophages consistent with impaired M1-to-M2 transition (1, 21). Aging, by contrast, features preserved MRF expression, paradoxical worsening upon TH replacement, and altered macrophage cytokine profiles rather than a simple polarization defect (20, 22). These distinctions motivate the comparison in Section 3.4.

Physiological regeneration proceeds through overlapping phases rather than discrete steps. An early degenerative-inflammatory phase clears damaged tissue, followed by MuSC activation and proliferation, a transition toward differentiation and early fiber formation, and later remodeling and maturation. MuSCs, fibro-adipogenic progenitors (FAPs), endothelial cells, neutrophils, macrophages, regulatory T cells, and extracellular-matrix components (ECM) participate at different times, so successful repair depends on both cell-intrinsic competence and coordinated intercellular signaling (23–27). Aging perturbs several elements of this sequence, raising the possibility that regenerative failure can arise from impaired integration of endocrine cues with the changing niche (26, 28–30).

Here, we aimed to determine whether published evidence and cross-dataset computational analyses are consistent with a model in which aging preserves parts of THR activation but disrupts their coordination with myogenic, stromal, and immune programs. We further aimed to distinguish this proposed integration defect from the primary reduction in endocrine activation expected in hypothyroidism and to define predictions that can be tested experimentally.

## 2 Endocrine-immune-niche integration: a new framework

We propose that aging disrupts the mechanisms that integrate endocrine signaling with immune responses and niche-derived regulatory programs, and that regenerative decline reflects progressive uncoupling of TH signaling from the cellular networks required for effective tissue repair. A central feature of this framework is the existence of two complementary THR-dependent regulatory axes. The first is a stromal axis linking THR activity to extracellular matrix organization and niche remodeling. FAPs are positioned at the center of this axis, translating endocrine signals into structural and paracrine programs that shape the regenerative environment. The second is a cross-lineage integration axis that coordinates THR activity across myogenic, stromal, and immune populations, embedding endocrine signals within broader networks of inflammatory resolution, intercellular communication, and regenerative timing. Under physiological conditions, these axes operate in a coordinated manner: THR activation is coupled to transcriptional execution, extracellular matrix remodeling, and appropriate communication between regenerative cell populations. Aging progressively weakens this coordination. Rather than producing complete loss of TH signaling, aging destabilizes the relationships linking receptor activation to downstream transcriptional programs and extracellular signaling networks. The novelty of this framework is that it shifts the focus from thyroid hormone receptor activity itself to the mechanisms that couple receptor activation with regenerative execution. Endocrine signaling remains necessary but not sufficient for regeneration; regenerative competence emerges from successful integration of endocrine, immune, and niche-derived inputs, whereas regenerative failure reflects progressive uncoupling among these regulatory layers. This model generates a testable prediction: aging should preserve substantial components of THR activation while selectively disrupting the transcriptional, stromal, and intercellular networks through which endocrine signals are translated into tissue repair. The following sections examine whether the available evidence is consistent with this prediction.

## 3 Evidence supporting impaired endocrine-immune-niche integration model

### 3.1 Evidence for uncoupling between THR activity and regeneration execution

We integrated single-cell transcriptomic, regulatory network, and chromatin accessibility datasets spanning skeletal muscle regeneration in young and aged mice (GSE143437, GSE232106, GSE121589) (31–34). Full dataset inclusion, quality-control, scoring, and statistical procedures are provided in the **Supplementary Methods** and summarized in the Data Sources and Analytical Framework section. THR activity was estimated with CollecTRI-VIPER, a regulon-based method that infers transcription-factor activity from coordinated behavior of curated target genes rather than receptor abundance alone (35, 36). Thus, a ‘regulon’ denotes a transcription factor together with its predicted or curated target genes, and the resulting score is a computational proxy rather than a direct measurement of receptor binding or activity.

**Figure 1:**
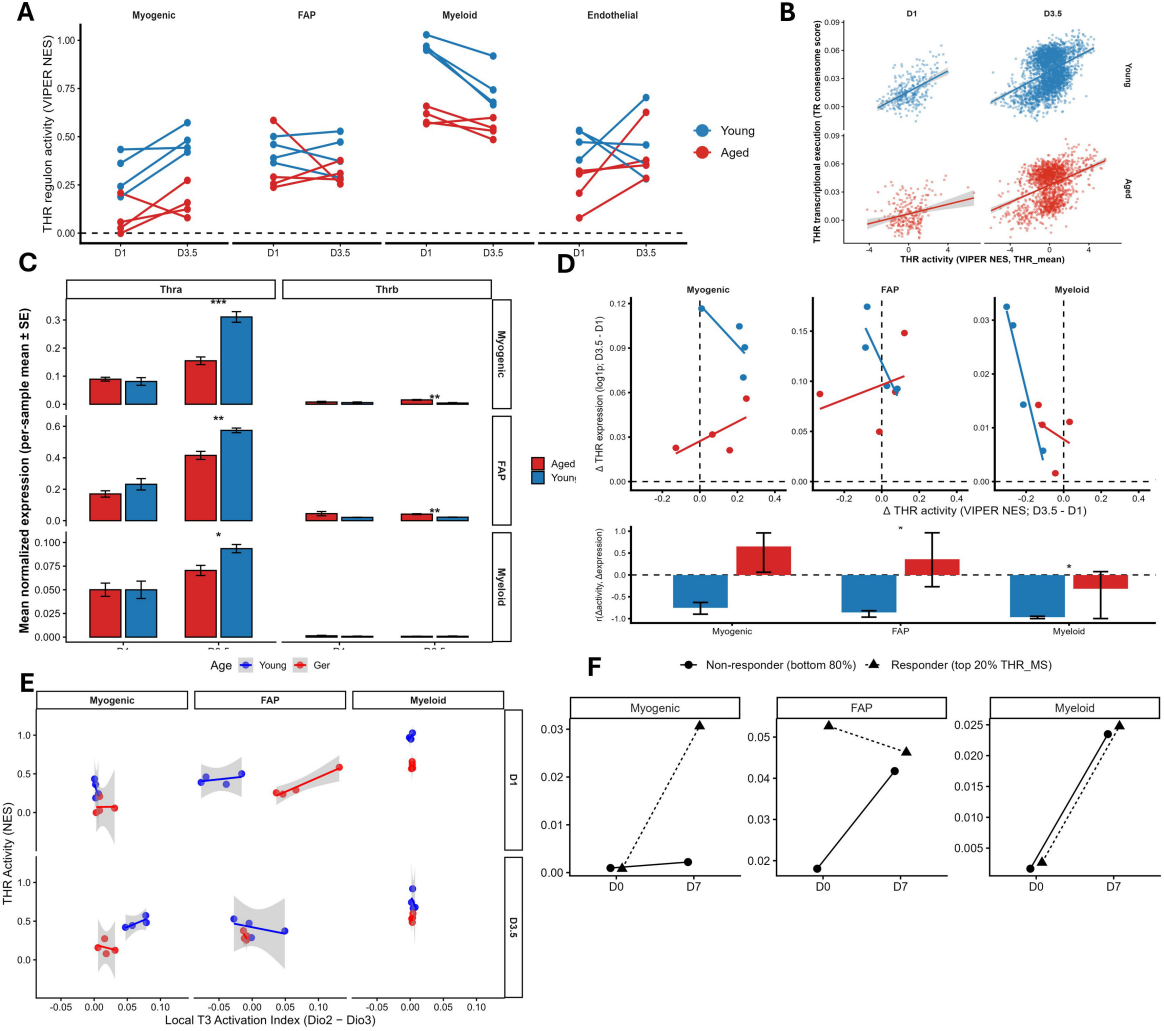
Aging disrupts coordination and transcriptional execution of THR signaling during muscle regeneration. **(A)** Lineage-resolved THR regulon activity inferred by CollecTRI-VIPER in myogenic, FAP, myeloid, and endothelial cells at day 1 (D1) and day 3.5 (D3.5) following injury in young (blue) and aged (red) mice. Each line represents an individual biological replicate; dashed line indicates baseline activity. **(B)** Association between inferred THR activity and transcriptional execution. THR regulon activity (CollecTRI-VIPER) is plotted against the THR consensome-based execution score (TRcons_1), computed from TC90 genes present in the dataset. Each point represents a biological replicate, stratified by age and time point. Solid lines indicate linear regression fits with 95% confidence intervals. The consistent positive association across conditions supports the validity of the consensome-derived gene set as a proxy for downstream THR transcriptional output. **(C)** Mean normalized expression of *Thra* and *Thrb* in myogenic, FAP, and myeloid lineages at D1 and D3.5. Bars represent per-sample means ± SEM. Statistical significance was assessed using two-sided Welch’s t-tests based on biological replicates (*p < 0.05, **p < 0.01, ***p < 0.001). **(D)** Relationship between changes in THR activity (ΔVIPER NES) and receptor expression (Δlog1p *Thra/Thrb*) from D1 to D3.5. Points represent biological replicates; lines indicate linear regression for young (blue) and aged (red) mice. Bar plots summarize Pearson correlations with leave-one-out cross-validation (LOOCV) ranges, illustrating destabilized coupling between receptor expression and activity in aged muscle. **(E)** Association between THR activity and local T3 activation index (*Dio2 - Dio3*) at D1 and D3.5. Points represent biological replicates; lines indicate linear regression with 95% confidence intervals, showing increased heterogeneity of TH-dependent metabolic regulation with aging. **(F)** Longitudinal changes in the T3 activation index from baseline (D0) to D7 in aged muscle, stratified by THR activity. Cells were classified by THR module score (THR_MS), with the top 20% defined as high-THR responders. High-THR cells show selective induction of T3 activation, indicating that local thyroid hormone metabolism operates in a THR-dependent, gated manner rather than reflecting uniform ligand availability.

The analysis showed lineage- and time dependent THR activity during regeneration (**Figure 1A**).

Young myogenic populations displayed a coordinated increase, whereas this temporal pattern was attenuated with aging; nevertheless, THR-associated activity remained detectable in several lineages. This observation is compatible with but does not prove tissue-level thyroid hormone resistance (13, 20, 37).

A more specific age-associated pattern involved the relationship between inferred THR activity and downstream transcriptional programs (**Figure 1B**; **Supplementary Figure S1**). Coupling to Myod1, a myogenic regulatory factor that promotes commitment and differentiation, was attenuated in aged samples (6–9), whereas relationships involving Foxo3, a stress- and metabolic-response regulator (3), were comparatively preserved. Myf5, one of the earliest myogenic regulatory factors induced during MuSC activation and a promoter of transient myoblast amplification during regeneration, provides additional biological context (38). The deiodinases Dio2 and Dio3, which respectively activate and inactivate thyroid hormone locally, regulate intracellular T3 availability and help control the balance between proliferation and differentiation (10, 18). Experimental studies report that THRA loss of function reduces MyoD1, Myf5, and mitochondrial-quality-control factors (14); persistent COUP-TFII can interfere with co-regulator recruitment at thyroid-response elements, meaning that receptor-associated transcription is altered without receptor deletion (2); and FoxO3 can induce Dio2 during myogenesis (3). Together, these findings make selective uncoupling biologically plausible, but the cross-dataset associations do not identify its molecular cause.

Receptor abundance and ligand metabolism did not fully account for this pattern. *Thra* expression was modestly reduced, whereas relationships involving the Dio2/Dio3 local T3-activation axis remained evident but more heterogeneous (**Figures 1C–F**) (10, 39). Chromatin-accessibility analysis likewise indicated that nuclear-receptor-enriched regions remained broadly accessible, although accessibility does not establish THR binding or productive transcription (**Supplementary Figures S2**, **S3**). This distinction is important because an open locus can still exhibit altered co-regulator use, enhancer-promoter communication, or transcriptional variability during aging (40, 41).

**Figure 2:**
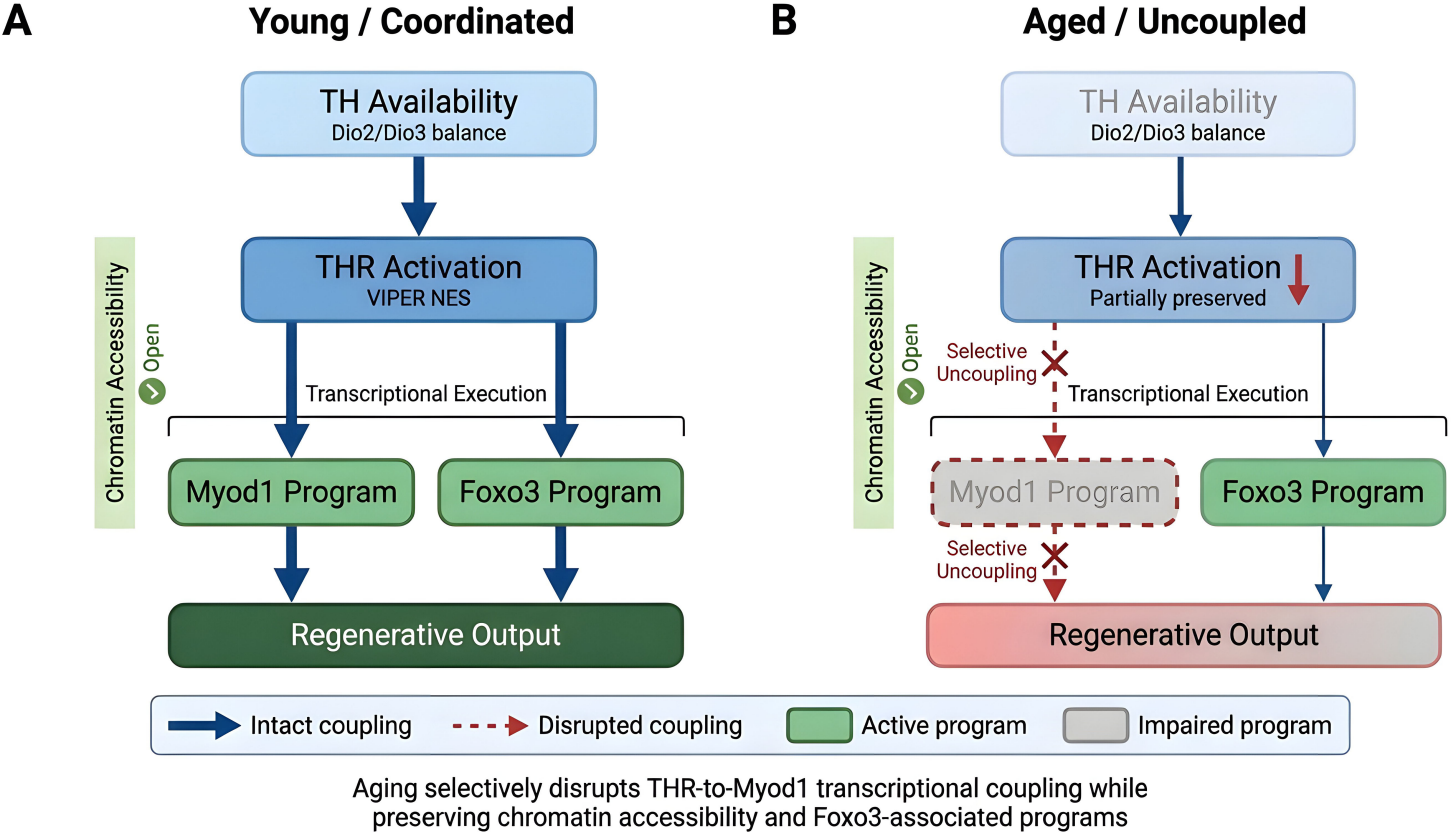
Schematic summary of age-dependent selective uncoupling between THR activation and transcriptional execution during skeletal muscle regeneration. **(A)** Young/Coordinated regeneration. Thyroid hormone (TH) availability, regulated by the balance between activating (Dio2) and inactivating (Dio3) deiodinases, drives THR activation (inferred by CollecTRI-VIPER normalized enrichment scores). In young muscle, THR activation is tightly coupled to downstream transcriptional execution of both Myod1-associated differentiation programs and Foxo3-associated metabolic programs, resulting in effective regenerative output. Chromatin at TH-responsive loci remains accessible, as assessed by ATAC-seq chromVAR analysis. Solid blue arrows indicate intact coupling between regulatory levels. **(B)** Aged/Uncoupled regeneration. In aged muscle, TH availability and THR activation are partially preserved, and chromatin accessibility at nuclear receptor-enriched regulatory regions is maintained. However, the coupling between THR activation and Myod1-dependent transcriptional programs is selectively disrupted (dashed red arrow), while Foxo3-associated regulatory relationships remain relatively intact. This selective uncoupling results in impaired regenerative output despite persistent endocrine signaling and open chromatin architecture. The data supporting each level of this schematic are presented in **Figures 1A–F** and **Supplementary Figures S1–S3**.

Overall, these analyses are consistent with partially preserved THR-associated activity and selective weakening of downstream execution rather than uniform pathway loss (**Figure 2**). Experimental perturbation of THR, its co-regulators, and niche signals in matched young and aged systems will be required to establish whether this uncoupling is causal.

### 3.2 Evidence of coordination between THR signaling and stromal and immune remodeling through intrinsic and cross-lineage regulatory axes

**Figure 3:**
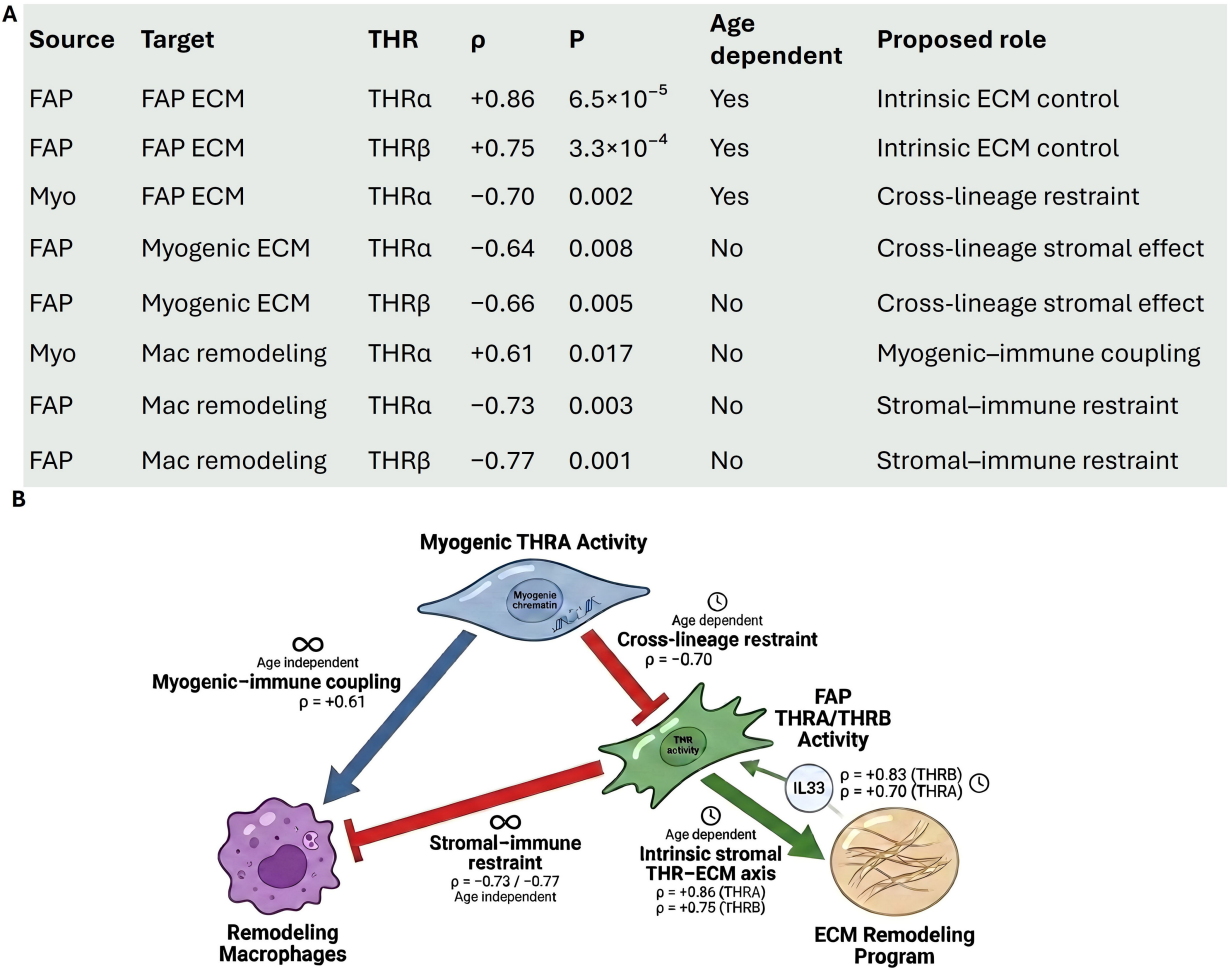
THR signaling coordinates stromal and immune remodeling through intrinsic and cross-lineage regulatory axes. **(A)** Summary of significant THR-associated relationships identified across regenerative compartments. Correlations were calculated at the replicate level using THR activity scores and compartment-specific remodeling programs. Positive and negative associations were classified according to directionality, and age dependence was determined using linear models incorporating age interaction terms. **(B)** Network representation of THR-associated interactions during regeneration. FAP THRA and THRβ activity were positively associated with extracellular matrix (ECM) remodeling programs, consistent with an intrinsic stromal THR–ECM axis. Myogenic THRA activity was negatively associated with FAP ECM programs, suggesting a cross-lineage regulatory relationship. Macrophage remodeling, defined by Apoe, Trem2, and Spp1 expression, was positively associated with myogenic THRA activity but negatively associated with FAP THR activity, consistent with myogenic–immune coupling and stromal–immune restraint. IL33 expression was positively associated with both THRA and THRB activity in FAPs, supporting a potential THR-regulated immune-regulatory stromal program. Age-dependent relationships are indicated by clock symbols.

Integration of THR-associated relationships across regenerative compartments revealed a structured network linking myogenic, stromal, extracellular matrix (ECM), and immune remodeling programs (**Figure 3**). ECM denotes the structural and signaling scaffold surrounding cells, including collagens, glycoproteins, and matrix-associated factors that influence adhesion, mechanics, and growth-factor availability. Within FAPs, THRA and THRB activity was positively associated with ECM transcriptional programs, whereas myogenic THRA activity was negatively associated with FAP ECM programs. These associations are compatible with stromal-intrinsic and cross-lineage THR-ECM axes, respectively, but they do not demonstrate direct signaling between the lineages. Supporting biological plausibility, D2-generated T3 influences FAP fate decisions (42), and COUP-TFII silencing in THRA-PV myoblasts reactivates ECM-related pathways (2).

Immune remodeling showed a distinct pattern. We used the mean normalized expression of Apoe, Trem2, and Spp1 as a replicate-level macrophage remodeling score (full definition in **Supplementary Methods**). These genes mark lipid handling and phagocytic adaptation (Apoe), damage-associated myeloid sensing (Trem2), and osteopontin-linked matrix and inflammatory remodeling (Spp1); the score therefore represents a remodeling-associated state rather than a universal M1/M2 identity. The score was positively associated with myogenic THRA activity and negatively associated with FAP THR activity. Experimental work showing that intracellular T3 regulation influences macrophage inflammatory function provides independent biological context (43, 44), but the direction of cross-lineage causation cannot be inferred from these correlations.

The Apoe/Trem2/Spp1 macrophage-score associations were largely age-independent, whereas the FAP THRA-IL33 relationship showed a significant age interaction (**Supplementary Figure S4**; **Figure 3B**). These results are not contradictory because they quantify different immune-related layers: the former is a macrophage-state readout whose relationship with THR was comparatively preserved, while the latter is a stromal signal relevant to recruitment and maintenance of regulatory T cells (Tregs). IL33 expression correlated with FAP THRA and THRB activity, and prior work identifies FAP-lineage cells as a major muscle IL-33 source and shows that IL-33 administration improves Treg accumulation and regeneration in aged mice (45, 46). We therefore interpret the THR-FAP-IL33 relationship as preliminary evidence of an age-sensitive stromal-immune interface, not as proof that THR directly controls IL33 or Treg behavior.

### 3.3 Aging reshapes how THR signaling is interpreted within the regenerative niche

A central prediction of the endocrine-immune-niche framework is that the biological consequences of THR activity depend not only on receptor activation itself but also on the extracellular signaling environment in which endocrine signals are interpreted. If this model is correct, aging should alter not only individual regenerative pathways but also the relationships linking endocrine signaling to intercellular communication networks.

**Figure 4:**
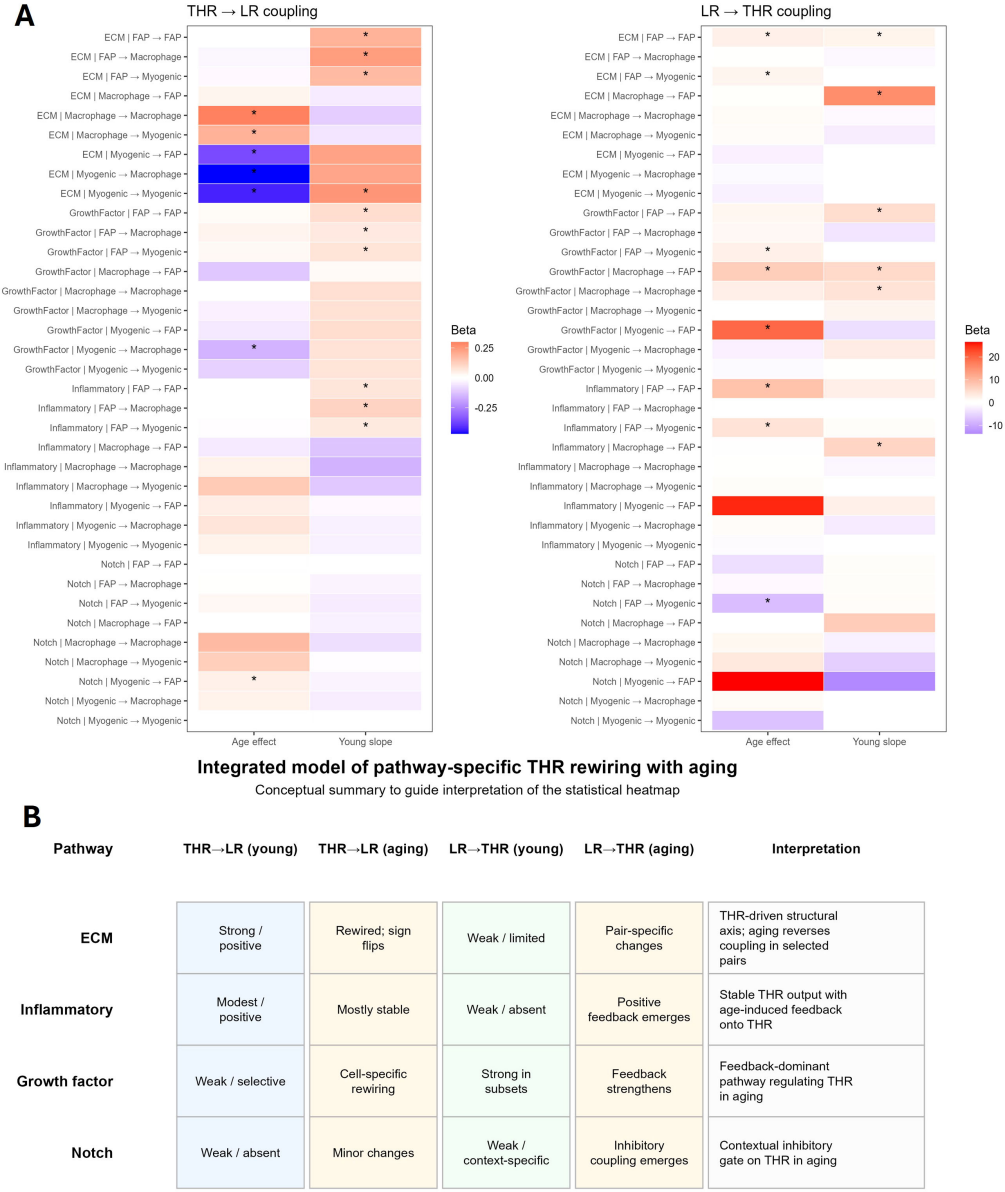
Age-dependent relationship between thyroid hormone receptor (THR) activity and niche communication networks. **(A)** Heatmap summary of pathway-specific associations between THR activity and ligand–receptor (LR) signaling programs across regenerative cell populations. Left panels show associations between THR activity and outgoing LR signaling modules (THR→LR coupling), whereas right panels show associations between incoming LR signaling modules and THR activity (LR→THR coupling). Color intensity represents model coefficients (β), and asterisks indicate statistically significant associations. Rows are organized by signaling pathway (ECM/Integrin, Growth Factor, Inflammatory, and Notch) and source–target cellular interactions. **(B)** Conceptual summary table highlights pathway-level patterns of age-dependent rewiring and their proposed biological interpretation.

**Figure 5:**
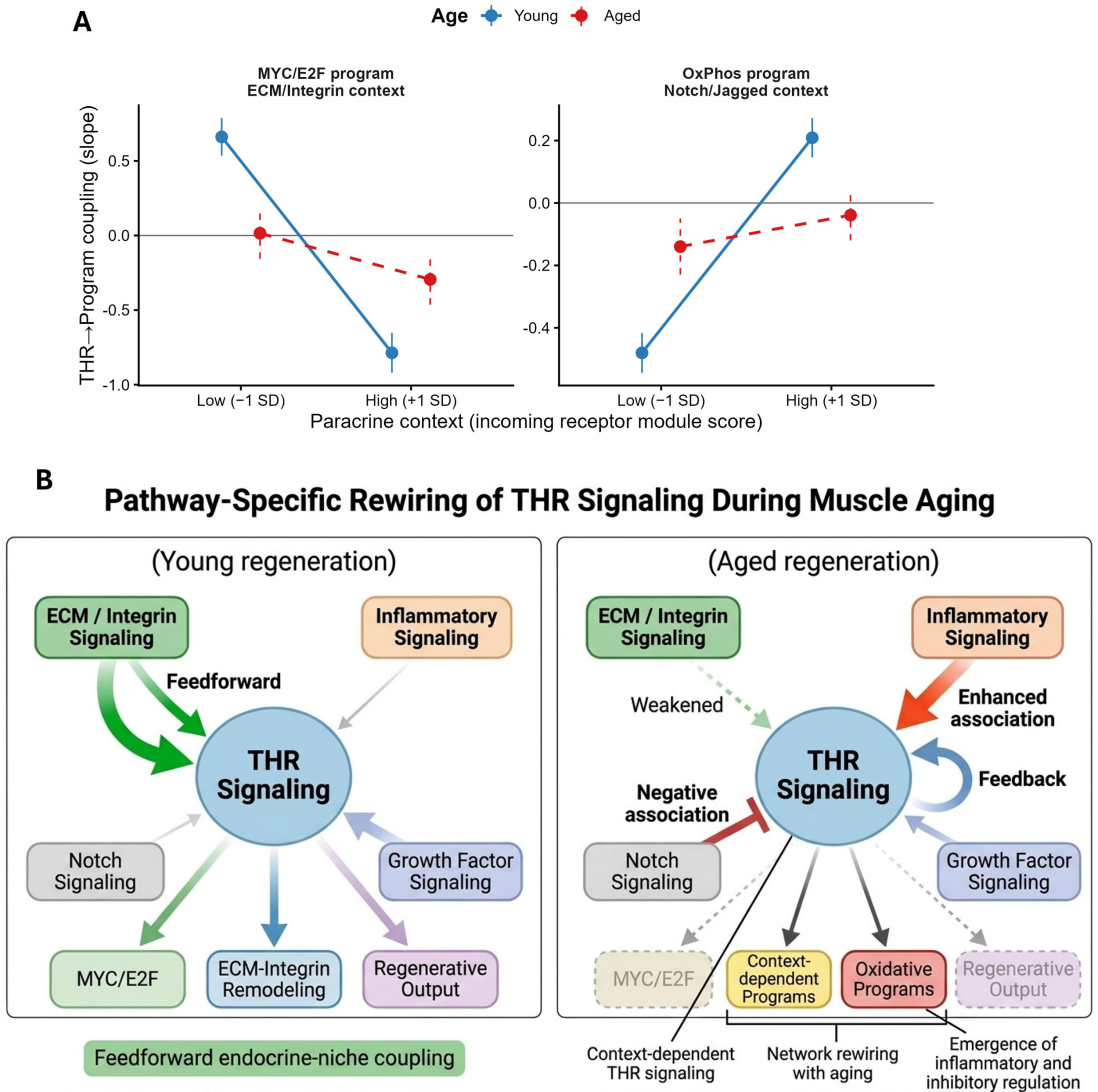
Context-dependent interpretation of THR activity during aging. **(A)** Estimated slopes of THR-program coupling under low versus high LR input for ECM (Myc/E2F program) and Notch (OxPhos program). Points represent model-derived marginal slopes; error bars indicate 95% confidence intervals. **(B)** Conceptual model in which aging weakens ECM-associated coupling, increases inflammatory and growth-factor dependencies, and alters Notch relationships. MYC/E2F denotes proliferation and cell-cycle programs; FOXO/PGC1α and oxidative programs denote stress adaptation and mitochondrial metabolism.

To examine this possibility, we analyzed ligand-receptor signaling pathways involved in regeneration and modeled their association with THR activity across regenerative cell populations (**Supplementary Figure S5**). This analysis revealed widespread, lineage- and stage-specific rewiring of the association between THR activity and transcriptional programs across ECM/Integrin, IGF1, and Notch/Jagged pathways (**Supplementary Figure S6**). Consistent with the proposed stromal axis, ECM signaling exhibited the strongest association with THR activity (**Figures 4A, B**). In FAPs, THR activity was positively associated with ECM signaling output, suggesting that endocrine signaling contributes to niche remodeling through stromal transcriptional programs. In myogenic cells, this relationship was positive in young muscle but became attenuated or inverted with aging, indicating disruption of the normal coupling between endocrine activity and extracellular matrix-associated regenerative responses. Moreover, ECM signaling was the strongest predictor of entry into a THR-high transcriptional state (**Figure 5A**). This ECM-endocrine coupling is consistent with proteomic evidence that aging extensively remodels the MuSC niche ECM, with FAP-derived matricellular proteins disrupting integrin-MAPK signaling axes critical for stem cell function (47).

In contrast to ECM signaling, inflammatory pathways displayed a distinct pattern of age-dependent rewiring. In young muscle, inflammatory signaling showed limited association with THR activity, whereas in aged muscle positive associations emerged between inflammatory pathway activity and THR signaling (**Figures 4A, B**). This is supported by multi-omic profiling showing that aged MuSCs undergo progressive enhancer rewiring that activates proinflammatory programs (48) and suggests that aging shifts endocrine signaling toward a more inflammation-dependent regulatory context, consistent with altered integration of immune and regenerative programs.

Growth-factor signaling provided a third context-dependent layer. IGF-family ligand-receptor activity was positively associated with THR output in selected sender-receiver relationships, and age interaction terms suggested stronger dependence of THR-associated programs on extracellular growth-factor input (**Figure 4**). Mechanistically, IGF signaling can activate PI3K-AKT and MAPK pathways that support MuSC survival, proliferation, and differentiation, while AKT-mediated inhibition of FOXO factors can shift the balance between catabolic/stress-response and growth programs. Because the analysis aggregates curated ligand-receptor expression at the replicate level, it identifies a candidate interface between IGF signaling and THR-associated transcription but cannot establish receptor engagement, pathway phosphorylation, or direction of causality.

Notch signaling displayed a particularly distinctive pattern. While Notch pathway activity showed minimal association with THR-dependent transcriptional programs in young muscle, aging was associated with the emergence of negative relationships between Notch signaling and THR activity, especially within FAP-to-myogenic interactions (**Figure 4**). Increased Notch signaling was also associated with a shift in THR-dependent transcriptional output away from proliferative programs and toward oxidative gene expression (**Figure 5A**). These observations are consistent with the established role of Notch signaling in maintaining progenitor identity and limiting differentiation (49, 50), suggesting that Notch may function as a contextual regulator that modifies how endocrine signals are translated into regenerative responses.

Collectively, these findings indicate that aging reorganizes the relationship between endocrine signaling and the extracellular communication networks that coordinate tissue repair. THR activity transitions from a predominantly feedforward-associated program in young muscle to a context-dependent system increasingly shaped by stromal, inflammatory, growth factor, and inhibitory signaling inputs (**Figure 5B**). This network-level rewiring provides additional evidence that age-associated regenerative decline reflects impaired endocrine-immune-niche integration.

### 3.4 Aging and hypothyroidism reveal distinct modes of endocrine dysfunction

A central implication of the endocrine-immune-niche integration framework is that aging should not simply represent a state of reduced thyroid hormone signaling. If age-associated regenerative decline were primarily driven by loss of endocrine activation, the regulatory architecture observed during aging would be expected to resemble that of hypothyroidism, a condition in which THR signaling is directly constrained by reduced hormone availability. We therefore compared aging-associated alterations in THR regulation with those previously identified in a hypothyroid regeneration model (GSE311479) (1).

**Figure 6:**
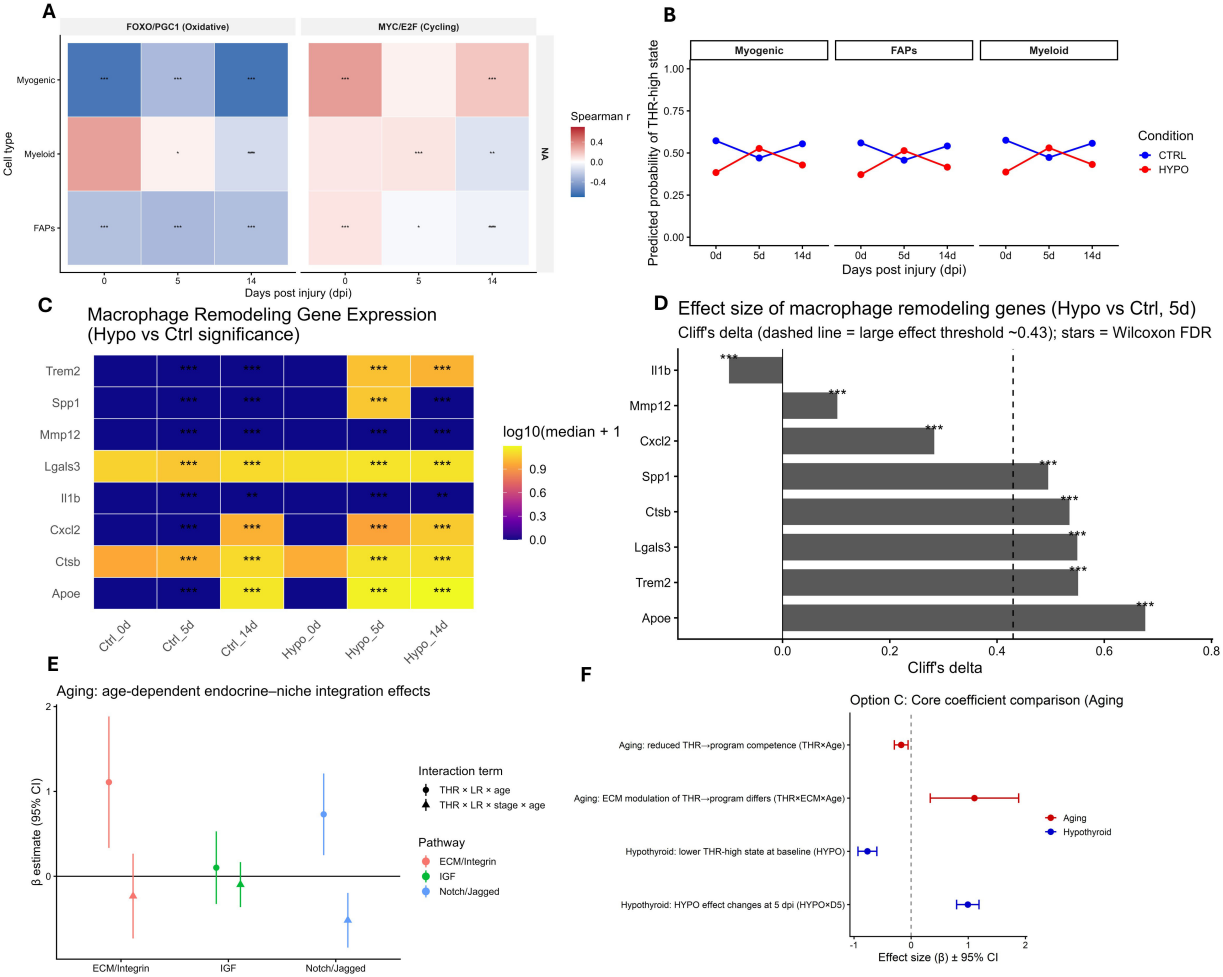
Distinct endocrine-niche mechanisms underlie impaired THR signaling in aging and hypothyroidism. **(A)** Heatmaps showing Spearman correlations between THR activity and FOXO/PGC1α (oxidative) and MYC/E2F (proliferative) transcriptional programs across cell types and regeneration time points (0, 5, and 14 days post-injury). Correlations are shown for control and hypothyroid conditions. Color scale indicates Spearman’s ϱ; asterisks denote statistical significance (*P < 0.05, **P < 0.01, ***P < 0.001). **(B)** Predicted probability of cells occupying a THR-high transcriptional state across regeneration stages in control and hypothyroid muscle, stratified by cell type. Values are derived from generalized linear mixed-effects models; lines indicate model-predicted means. **(C)** Expression of macrophage remodeling-associated genes in control and hypothyroid muscle. Heatmap shows median expression of remodeling-associated macrophage markers (Apoe, Trem2, Spp1, Lgals3, Ctsb, Mmp12, Cxcl2, Il1b) across regeneration stages. Asterisks indicate significant differences between hypothyroid and control conditions. Hypothyroidism promotes sustained activation of a remodeling-associated macrophage program during regeneration. **(D)** Effect sizes for macrophage remodeling genes at day 5 post injury. Bars show Cliff’s delta values comparing hypothyroid and control myeloid cells. Apoe, Trem2, Spp1, Lgals3, and Ctsb exhibit large positive effect sizes, indicating robust expansion of remodeling-associated macrophage states in hypothyroid muscle. The dashed line denotes the threshold for a large effect size (|δ| ≈ 0.43). **(E)** Pathway-specific interaction coefficients from mixed-effects models evaluating endocrine-niche integration in aging. Points represent β estimates and error bars indicate 95% confidence intervals for THR x LR x age and THR x LR x stage x age interaction terms across ECM/Integrin, IGF, and Notch/Jagged pathways. Colors indicate signaling pathways and point shapes denote interaction types. **(F)** Comparison of key regulatory coefficients between aging and hypothyroidism models. Shown are terms capturing intrinsic THR-program competence (THR x age), ECM-dependent modulation (THR x ECM x age), baseline hypothyroid effects, and time-dependent hypothyroid responses at 5 days post-injury. Points represent β estimates and horizontal bars indicate 95% confidence intervals. Vertical dashed line denotes no effect (β = 0).

In control muscle, THR activity was associated with major regenerative transcriptional programs across several lineages, including MYC/E2F networks, which broadly represent cell-cycle entry and proliferation, and FOXO/PGC1α-associated networks, which represent stress adaptation, oxidative metabolism, and mitochondrial regulation (**Figure 6A**). Hypothyroidism disrupted these relationships, producing attenuation and temporal delay of THR-dependent transcriptional programs. Consistent with a primary defect in endocrine activation, hypothyroidism significantly reduced the probability of entering a THR-high transcriptional state across myogenic, stromal, and immune compartments (**Figure 6B**). These results are consistent with our recent experimental data (1), while remaining subject to the limitations of cross-dataset comparison.

We next examined whether immune remodeling programs were similarly affected in hypothyroid regeneration. Consistent with previous observations of persistent inflammation, hypothyroid muscle exhibited marked induction of a remodeling-associated macrophage state during the mid-regenerative phase. At day 5 post-injury, expression of Apoe, Trem2, and Spp1 was substantially increased relative to controls, with large effect sizes (Cliff’s δ = 0.68, 0.55, and 0.50, respectively) and 8-15-fold increases in median expression (FDR < 10^-122^). Expression of Spp1 and Apoe remained strongly correlated at the single-cell level in both control and hypothyroid conditions, consistent with coordinated activation of a macrophage remodeling program. Although expression differences persisted at day 14, effect sizes were attenuated, suggesting delayed rather than permanently arrested resolution. These findings indicate that hypothyroidism promotes sustained activation of Spp1^+^/Trem2^+^/Apoe^+^ remodeling macrophages during regeneration (**Figure 6C**). Notably, this Spp1^+^/Trem2^+^ macrophage signature has been identified as a conserved pro-fibrotic polarization state across multiple organs, where these cells drive ECM deposition through osteopontin-CD44 signaling and fibroblast-to-myofibroblast transdifferentiation (51). In contrast, aging was characterized by relative preservation of major THR–macrophage associations despite disruption of THR-dependent stromal and cross-lineage coordination (**Figure 3**).

Aging produced a fundamentally different pattern. Rather than causing a uniform reduction in THR activation, aging selectively altered the relationship between THR activity and niche-derived regulatory inputs (**Figure 6D**). Positive THR × ligand-receptor × age interaction terms were particularly evident for ECM/Integrin and Notch/Jagged pathways, indicating that THR-dependent transcriptional output became increasingly dependent on extracellular signaling context. Additional higher-order interactions revealed extensive temporal remodeling of these relationships throughout regeneration.

Direct comparison of model coefficients further highlighted these mechanistic differences (**Figure 6E**). Aging was characterized by reduced intrinsic THR-program coupling together with enhanced dependence on extracellular signaling inputs, consistent with preserved receptor activation but unstable transcriptional execution. In contrast, hypothyroidism was dominated by reduced THR activation and diminished entry into THR-high transcriptional states, indicating a primary defect at the level of hormone availability.

**Figure 7:**
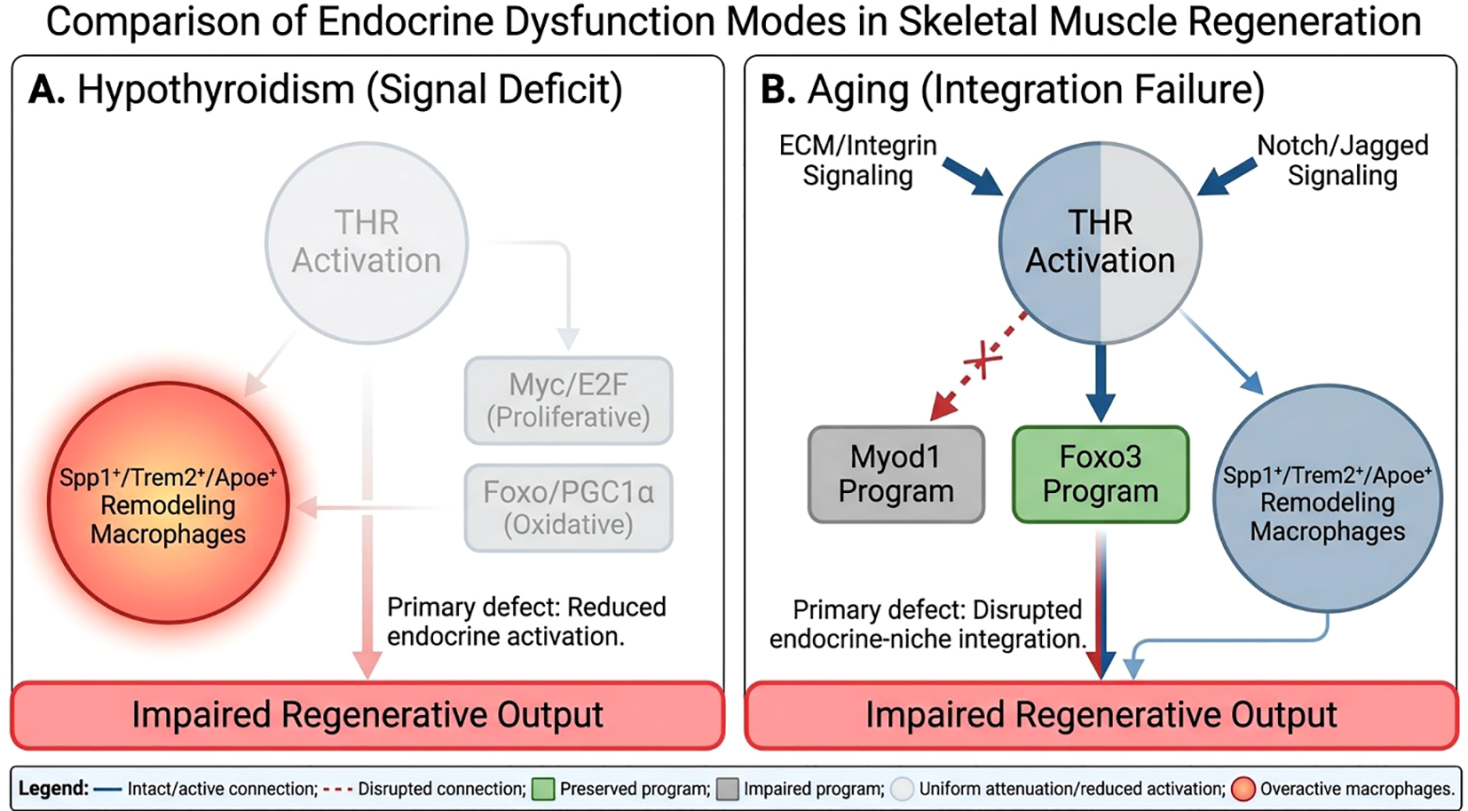
Comparison of Endocrine Dysfunction Modes in Skeletal Muscle Regeneration. **(A)** In hypothyroidism, reduced hormone availability uniformly attenuates THR activation (light gray circle), globally suppressing downstream Myc/E2F and Foxo/PGC1α programs while promoting sustained overactivation of Spp1^+^/Trem2^+^/Apoe^+^ remodeling macrophages (enlarged red circle). The primary defect is reduced endocrine activation. **(B)** In aging, THR activation is preserved (filled blue circle) but becomes context-dependent, increasingly shaped by ECM/Integrin and Notch/Jagged inputs. Downstream coupling is selectively disrupted: the THR–MyoD1 axis is impaired (dashed red line; gray box) while THR–Foxo3 (green box) and THR–macrophage remodeling associations remain relatively intact. The primary defect is disrupted endocrine-niche integration rather than signal deficit. Solid blue lines = intact connections; dashed red lines = disrupted connections; green boxes = preserved programs; gray boxes = impaired programs. This schematic synthesizes computational findings from **Figure 6**.

Together, these observations suggest that hypothyroidism and aging impair regeneration through distinct mechanisms: hypothyroidism primarily alters endocrine activation and immune remodeling states, whereas aging predominantly disrupts the integration of endocrine signaling with niche-dependent regulatory programs. **Figure 7** summarizes this comparison. This interpretation provides a conceptual explanation for why restoration of thyroid hormone availability alone may be insufficient in aging.

## 4 Discussion

The analyses presented here provide converging support for the endocrine-immune-niche integration framework proposed in this study. Four associative patterns motivate the model. First, THR activity remains detectable across multiple regenerative lineages in aged muscle, yet its coupling to downstream transcriptional programs, particularly Myod1-associated differentiation, becomes selectively attenuated (**Supplementary Figure S1**; **Figure 2**). This dissociation between receptor activation and transcriptional execution cannot be explained by simple receptor deficiency or epigenetic silencing, as both *Thra* expression and chromatin accessibility at TH-responsive loci are at least partially preserved (**Supplementary Figures S2**, **S3**). Second, THR activity is embedded within a structured multicellular regulatory network involving intrinsic stromal control (FAP THR-ECM axis), cross-lineage communication (myogenic THR-FAP ECM restraint), and endocrine-immune coordination (THR-macrophage remodeling associations) (**Figure 3**). Aging selectively perturbs the stromal components of this network while preserving portions of the endocrine-immune axis. Third, the relationship between THR activity and intercellular signaling pathways undergoes extensive age-dependent rewiring: ECM coupling inverts, inflammatory and growth factor dependencies increase, and Notch signaling acquires inhibitory relationships with THR-dependent transcriptional output (**Figures 4**, **5**). Fourth, comparison with hypothyroidism reveals distinct regulatory patterns (**Figures 6**, **7**). Importantly, each of these major computational observations is supported by independent experimental studies spanning thyroid hormone biology, skeletal muscle regeneration, aging, immune remodeling, and niche regulation (**Supplementary Table 1**). The convergence between computational inference and previously established experimental findings strengthens the biological plausibility of the proposed endocrine-immune-niche integration framework.

### 4.1 Relationship to the cell-intrinsic model

**Figure 8:**
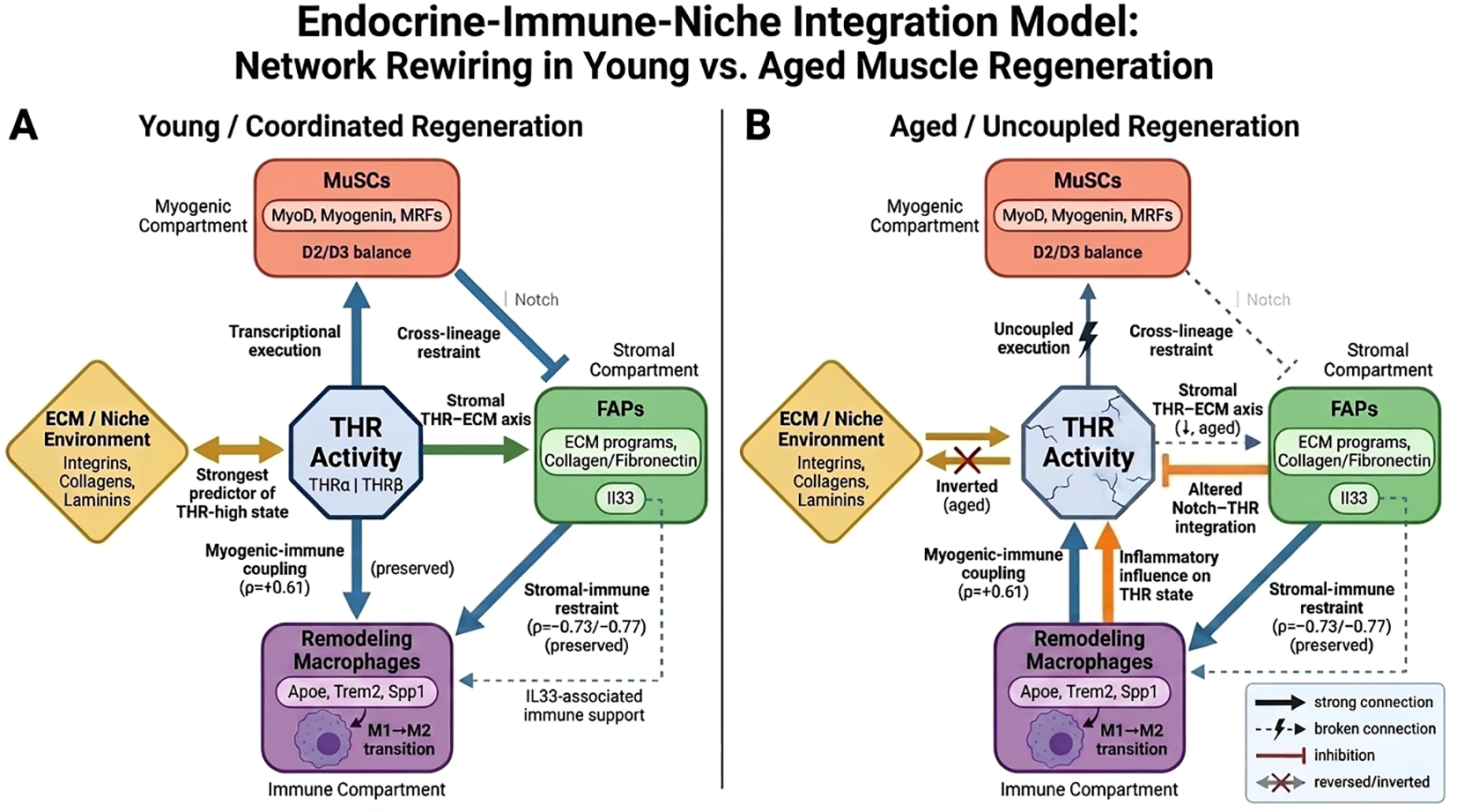
Endocrine-immune-niche integration model of network rewiring during young and aged skeletal muscle regeneration. Conceptual framework summarizing the relationships identified between thyroid hormone receptor (THR) activity, extracellular matrix (ECM) programs, stromal niche components, and immune remodeling during muscle regeneration. **(A)** Young/coordinated regeneration. THR activity functions as an integrated signaling hub linking myogenic, stromal, immune, and ECM compartments. THR activity is associated with execution of myogenic transcriptional programs, regulation of FAP ECM programs, and coordinated communication between regenerative compartments. ECM-associated pathways represent the strongest predictors of the THR-high state. FAP THR activity is positively associated with ECM remodeling programs and IL33 expression, whereas myogenic THR activity exhibits cross-lineage restraint of FAP ECM programs. THR activity is also linked to remodeling macrophage states characterized by Apoe, Trem2, and Spp1 expression. Stromal–immune restraint and myogenic–immune coupling are preserved, supporting coordinated progression through regenerative macrophage transitions. **(B)** Aged/uncoupled regeneration. Aging is proposed to preserve selected THR-dependent programs while disrupting integration across regenerative compartments. THR activity becomes partially uncoupled from downstream transcriptional execution and ECM-associated regulation. Age-dependent inversion of THR–ECM relationships, altered Notch–THR integration, and emergence of inflammatory influences on THR state contribute to network rewiring. In contrast, selected immune associations remain preserved, including myogenic–immune coupling and stromal–immune restraint. FAP-derived IL33 remains linked to immune-support pathways, but the overall endocrine–immune–niche network exhibits reduced coordination. Solid arrows indicate preserved or strong relationships supported by experimental analyses. Dashed arrows indicate weakened, altered, or inferred interactions. Inhibitory connections denote negative associations, whereas directional arrows indicate positive associations or predicted regulatory influence. THR, thyroid hormone receptor; MuSC, muscle stem cell; FAP, fibro-adipogenic progenitor; ECM, extracellular matrix.

The endocrine-immune-niche framework complements, rather than replaces, established intrinsic mechanisms in muscle stem cells (MuSCs or satellite cells) Aging reduces MuSC activation and proliferation, increases senescence-like and stress-associated states, alters epigenetic regulation and cell identity, and impairs self-renewal and differentiation (32, 34, 40, 48). The regulation of MyoD by THRA (7–9), the D2/D3-dependent control of intracellular T3 availability (3, 10, 11), and the requirement for THR in satellite cell maintenance (9) remain essential components of regeneration. Rather, the present analyses suggest that these cell-intrinsic mechanisms represent one layer of broader regulatory architecture. THR signaling may function less as a classical linear pathway and more as an organizational node that coordinates communication across cellular compartments. The conceptual framework summarizing these interactions is presented in **Figure 8**, in which THR activity occupies a central position within a network of reciprocal interactions among MuSCs, FAPs, remodeling macrophages, extracellular matrix programs, and immune-support pathways.

### 4.2 Reinterpretation of thyroid hormone replacement failure

The limited ability of thyroid hormone supplementation to restore regeneration in aged muscle has previously been interpreted as evidence that TH signaling is not a major determinant of regenerative aging (20). The present framework offers an alternative explanation: if endocrine signaling remains present but becomes uncoupled from stromal and immune execution programs, increasing hormone availability alone would be insufficient to restore regenerative competence. Regeneration fails not because endocrine signaling is absent, but because the tissue loses the ability to appropriately interpret endocrine information. This interpretation is consistent with the observation that T4 replacement in aged mice paradoxically worsened regenerative outcomes, including decreased cross-sectional area and increased cell death (20).

### 4.3 Immune implications beyond macrophages and contribution to understanding thyroid hormone-immune interactions

Macrophages were used as a tractable immune readout, but muscle repair depends on a broader and temporally ordered immune response. Neutrophils arrive early, release proteases and reactive mediators, and can also deliver pro-resolving signals that influence subsequent macrophage states; excessive or persistent neutrophil activity can damage tissue and impair MuSC function (52). Tregs accumulate later, promote macrophage transition and MuSC activation, and can support repair through amphiregulin; age-related loss of the FAP-derived IL-33 signal reduces local Treg accumulation (45, 46). Conventional T cells, natural killer cells, and other lymphoid populations may further shape cytotoxicity, cytokine tone, and resolution, although their relationships to THR signaling in regenerating muscle remain insufficiently defined (52). These populations could participate in the framework either as direct TH-responsive cells or as downstream recipients of THR-dependent stromal and myogenic signals.

Alternative, partially TH-independent aging mechanisms must also be considered. Cellular senescence, chronic low-grade inflammation, mitochondrial dysfunction, loss of proteostasis, epigenetic drift, and stem-cell exhaustion can impair regeneration without requiring an initiating defect in thyroid hormone signaling (40, 47, 48, 53). Senescent FAPs create an inflamed niche that restricts stem-cell proliferation and recruit macrophages through CCL2- and SPP1-associated programs (53), while macrophage-derived Selenoprotein P can impair inflammatory resolution (54). These processes may act upstream of THR uncoupling, downstream of it, or in parallel. The present analyses cannot distinguish among these possibilities; factorial experiments manipulating THR signaling and specific aging hallmarks will be needed.

Evidence also supports direct thyroid hormone effects on immune cells: intracellular T3 regulation is required for optimal macrophage inflammatory function (43, 44), hypothyroidism can alter galectin-1-producing Treg states with enhanced immunosuppressive capacity (55), and thyroid hormone has been linked to macrophage maturation and inflammatory responses (21). The THR-FAP-IL33 axis identified in Section 3.2 provides a potential mechanism linking endocrine signaling to Treg recruitment during regeneration. Because Tregs contribute to both macrophage polarization and satellite cell activation (45).

However, evidence for direct THR control of neutrophils and broader lymphoid populations during muscle repair is limited. Therefore, the framework proposes that direct hormone-immune effects and indirect stromal or myogenic effects may interact within the tissue environment, rather than asserting that TH directly regulates every immune population. This perspective provides a conceptual bridge between thyroid hormone biology, immune regulation, and regenerative aging.

### 4.4 Human relevance and translational boundaries

Human evidence links thyroid status to muscle phenotypes but does not yet test the proposed regenerative network. In older euthyroid cohorts, lower thyroid hormones have been associated with lower muscle mass, poorer physical performance, or longitudinal decline in handgrip strength, although findings vary across outcomes and observational designs cannot establish causality (12, 56–58). Human muscle transcriptomic and single-nucleus studies also identify age-associated changes in myogenesis, mitochondrial programs, senescence, and immune composition (59, 60). These observations support translational plausibility, not validation: circulating hormone measures do not report local TH metabolism, receptor activity, or post-injury endocrine-immune coordination. This type of cross-dataset analysis is more tractable in murine models because genetically similar animals can be studied using standardized injury protocols, controlled endocrine conditions, matched regeneration time points, and multiple biological replicates within each experimental group. Comparable human analyses are complicated by interindividual variation in age, sex, comorbidities, medications, physical activity, thyroid status, biopsy site, and sampling conditions, as well as the limited feasibility of repeated muscle sampling across defined post-injury stages. Consequently, existing human datasets may lack the matched temporal structure and biological replication required to distinguish endocrine-niche coupling from interindividual and cohort-specific effects. Prospective human studies combining thyroid phenotyping with standardized muscle biopsy or exercise-recovery paradigms, repeated sampling where ethically feasible, and cell-resolved profiling will be required.

### 4.5 Broader implications

The endocrine-immune-niche integration framework may have implications beyond skeletal muscle. Aging affects multiple endocrine systems, including growth hormone, insulin-like growth factor, glucocorticoid, and sex steroid signaling, and in many cases, age-associated dysfunction is characterized not by complete loss of hormone signaling but by altered tissue responsiveness (20). The possibility that endocrine aging reflects progressive disruption of signal integration rather than simple hormone deficiency may extend to other tissues and physiological systems. Skeletal muscle regeneration provides a tractable model in which these principles can be explored experimentally.

### 4.6 Predictions and experimental tests

The framework generates several experimentally testable predictions: 1. *FAP-specific THR disruption.* Conditional deletion of *Thra/Thrb* in FAPs (e.g., using Pdgfra-CreERT2 drivers) should impair extracellular matrix coordination and downstream immune recruitment without abolishing myogenic THR activity, producing a phenotype distinct from global hypothyroidism. 2. *Niche restoration.* Restoration of a youthful niche environment, through heterochronic parabiosis, young FAP transplantation, or senolytic treatment, should improve coupling between THR activity and regenerative programs in aged muscle, even without exogenous thyroid hormone supplementation. 3. *Combinatorial therapy.* Therapies targeting both endocrine signaling and niche restoration (e.g., T3 combined with anti-inflammatory or anti-fibrotic interventions) should outperform thyroid hormone replacement alone in restoring regenerative competence. 4. *Contextual pathway manipulation*. Modulation of Notch, inflammatory, or extracellular matrix pathways should alter the context-dependency of THR transcriptional output. Specifically, Notch inhibition in aged muscle should shift THR-dependent programs from oxidative toward proliferative gene expression, providing a direct test of the proposed integration model.

These predictions distinguish the endocrine-immune-niche integration model from the traditional cell-intrinsic framework: the former predicts that niche-targeted interventions can restore THR-regenerative coupling without changing receptor activation, whereas the latter predicts that only increased THR activation or hormone availability will improve regeneration.

## 5 Limitations

Several limitations should be considered when interpreting these findings. First, all analyses are based on murine datasets; human skeletal muscle aging involves additional complexity including differences in immune composition, hormonal milieu, and regenerative capacity. Second, the analyses presented here are correlational and associative. Cross-lineage correlations, regulon scores, ligand-receptor products, and interaction models do not demonstrate ligand-receptor engagement, receptor occupancy, molecular directionality, or causation. Third, biological replicate numbers are limited (n = 4 per condition in the primary aging dataset), and although robustness was assessed through leave-one-out cross-validation and bootstrap resampling, replicate-level correlations and higher-order interaction models should be interpreted as estimates of directional association rather than precise effect-size measurements. Fourth, although the framework incorporates multiple dimensions of endocrine-immune-niche interactions, including macrophage remodeling states, IL33-associated stromal programs, and inferred immune-support pathways, direct characterization of the full immune landscape remains limited. The current analyses do not comprehensively evaluate additional immune populations such as regulatory T cells, neutrophils, or broader lymphoid compartments. Future studies incorporating higher-resolution immune profiling and direct assessment of endocrine–immune interactions will be necessary to more fully define how thyroid hormone signaling shapes immune regulation during regenerative aging. Fifth, THR activity was inferred computationally using the CollecTRI-VIPER framework rather than measured directly; while this approach captures functional signaling output, it remains a proxy that should be validated through complementary experimental approaches. Sixth, the comparison between aging and hypothyroidism was performed across independent datasets with different experimental designs, and direct head-to-head comparisons within a single experimental system would strengthen the conclusions. Finally, senescence, inflammaging, mitochondrial dysfunction, epigenetic drift, and other hallmarks of aging may generate the observed associations independently of TH signaling. The framework should therefore be viewed as a testable synthesis rather than a demonstrated causal mechanism.

## 6 Conclusion

The evidence presented here supports a model in which aging impairs skeletal muscle regeneration partially through disrupted endocrine-immune-niche integration rather than a uniform loss of THR signaling. This framework links persistent THR activity in aged tissues, limited efficacy of thyroid hormone replacement, and distinct patterns of aging and hypothyroidism, and generates experimentally testable predictions regarding endocrine-immune communication during tissue repair. As senescent cells, immune dysfunction, and niche remodeling are increasingly recognized as interconnected drivers of age-related regenerative decline, the endocrine-immune-niche integration framework offers a conceptual foundation for developing combinatorial therapeutic strategies that restore signaling coordination rather than targeting individual pathways in isolation.

## 7 Data sources and analytical framework

The framework proposed in this article was developed through integration of publicly available transcriptomic and chromatin accessibility datasets spanning skeletal muscle regeneration, aging, and hypothyroidism. The analyses were designed to evaluate specific predictions derived from the proposed hypothesis rather than to generate hypothesis-free discoveries. Thyroid hormone receptor (THR) activity, transcriptional programs, chromatin accessibility, stromal remodeling states, and intercellular signaling networks were analyzed across independent datasets and integrated at the biological replicate level to identify systems-level regulatory patterns associated with regenerative aging. Detailed dataset descriptions, analytical procedures, statistical models, robustness analyses, and computational workflows are provided in the **Supplementary Methods**.

## Data Availability

The datasets presented in this study can be found in online repositories. The names of the repository/repositories and accession number(s) can be found in the article/**Supplementary Material**.
