# Supplementary material for: Beyond hormone deficiency: a framework for endocrine-immune-niche integration in aging skeletal muscle regeneration
Source: Front Immunol. 2026 Jul 29;17:1906969. doi: 10.3389/fimmu.2026.1906969 (PMC13462458; doi:10.3389/fimmu.2026.1906969)
Supplement: Supplementary file 1 [file DataSheet1.pdf]

**SUPPLEMENTARY INFORMATION**

# Beyond Hormone Deficiency: A Framework for Endocrine-Immune-Niche Integration in Aging Skeletal Muscle Regeneration

Laura Perin[1,2], Stefano da Sacco[1,2] , Yan-Yun Liu[3], Gregory A Brent[3], Anna Milanesi[3]

## Supplemental Figures

**Figure S1. Relationship between THR regulon activity and Foxo3/Myod1 transcriptional programs. (A)** THR regulon activity (mean VIPER NES of Thra and Thrb) was correlated with VIPER-inferred TF regulon activity (Foxo3 or Myod1) across biological replicates (n=4 per age group). Points show the absolute Pearson correlation (|r|) within each age group (Young, Ger). Error bars denote the range of correlations obtained by LOOCV, reflecting the sensitivity of the estimated correlation to removal of a single replicate. **(B)** Scatter plots show the relationship between THR regulon activity (mean VIPER normalized enrichment score [NES] of *Thra* and *Thrb*) and transcription factor regulon activity (VIPER NES for *Foxo3* or *Myod1*) in myogenic cells at day 3.5 after injury. Each point represents one biological replicate (n = 4 per age group). Solid lines indicate linear regression fits within each age group. Pearson correlation coefficients (r) and LOOCV ranges are shown within each panel to illustrate the strength and stability of the association. **(C)** Permutation-based null analysis of TF-THR correlations. Histograms show null distributions of Pearson correlation coefficients between TF regulon activity (Foxo3 or Myod1) and THR regulon activity across biological replicates, generated by randomly permuting TF activity values across replicates while preserving the original THR values (10,000 permutations per condition). Analyses were performed separately for young and geriatric muscle. Vertical black lines indicate the observed correlation computed using the true replicate pairing. In all cases, observed correlations fall in the extreme tails of the null distributions, indicating that TF-THR coupling is unlikely to be explained by chance replicate pairing. These permutation tests assess

the robustness of observed correlations given limited replicate number and do not imply causality. Lines connect correlations across time within each age group. The horizontal line indicates r = 0.

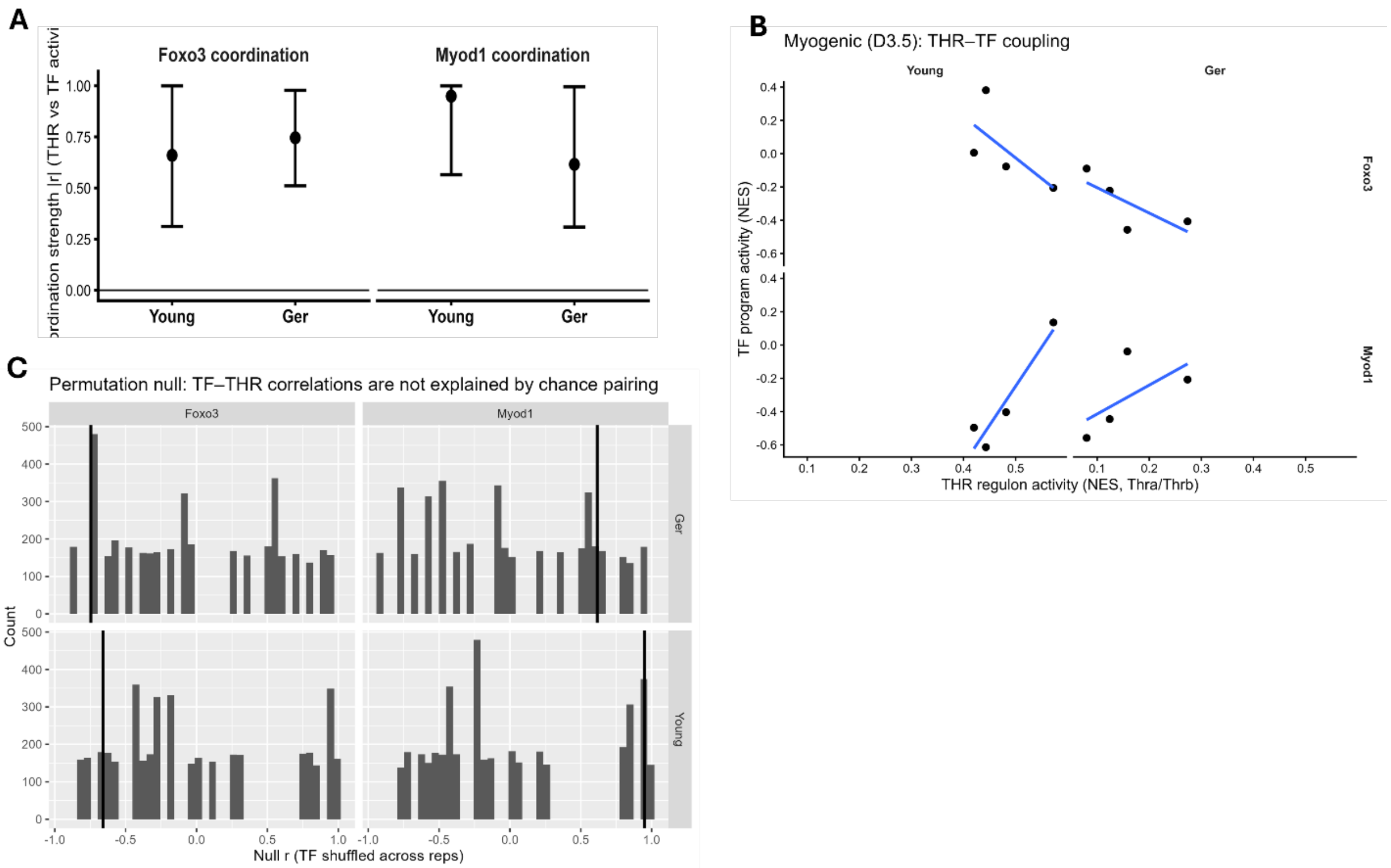


**Figure S2. Age-dependent transcriptional and chromatin divergence during muscle regeneration. (A-B)** Volcano plots showing differential gene expression between Aged and Young MuSCs at baseline (Day 0) and during regeneration (Day 5 post-injury). Differential expression analysis was performed using DESeq2 with Wald testing. Log2 fold changes were shrunk using the apeglm method to reduce variance inflation in low-count genes. For visualization, genes with mean normalized counts (baseMean) < 5 were excluded. Points are colored as follows: red, significantly upregulated in aged muscle (FDR < 0.05); blue, significantly upregulated in young muscle (FDR < 0.05); gray, not significant. Log2 fold changes are capped at ±8 for

display purposes only. The y-axis represents -log10 adjusted p-value (Benjamini–Hochberg FDR). **(C)** Quantification of differentially expressed genes (DE genes, FDR < 0.05) between aged and young MuSCs across the regeneration time course (Days 0, 1, 5, 7, and 21 post-injury). Differential expression was assessed at each time point using DESeq2 with Wald testing. Log2 fold changes were stabilized using apeglm shrinkage, while statistical significance was determined using Benjamini-Hochberg adjusted p-values. The number of DE genes peaks at Day 5, indicating maximal transcriptional divergence between aged and young MuSCs during early regenerative expansion. **(D)** Quantification of differentially expressed genes (RNA-seq; DE genes, FDR < 0.05) and differentially accessible chromatin regions (ATAC-seq; DA peaks, FDR < 0.05) between aged and young MuSCs across the regeneration time course. RNA divergence peaks at Day 5, indicating maximal transcriptional separation during early regenerative expansion. In contrast, chromatin accessibility differences are most pronounced at baseline (Day 0) and during later remodeling phases (Day 7), suggesting temporally distinct epigenomic and transcriptional responses to aging.

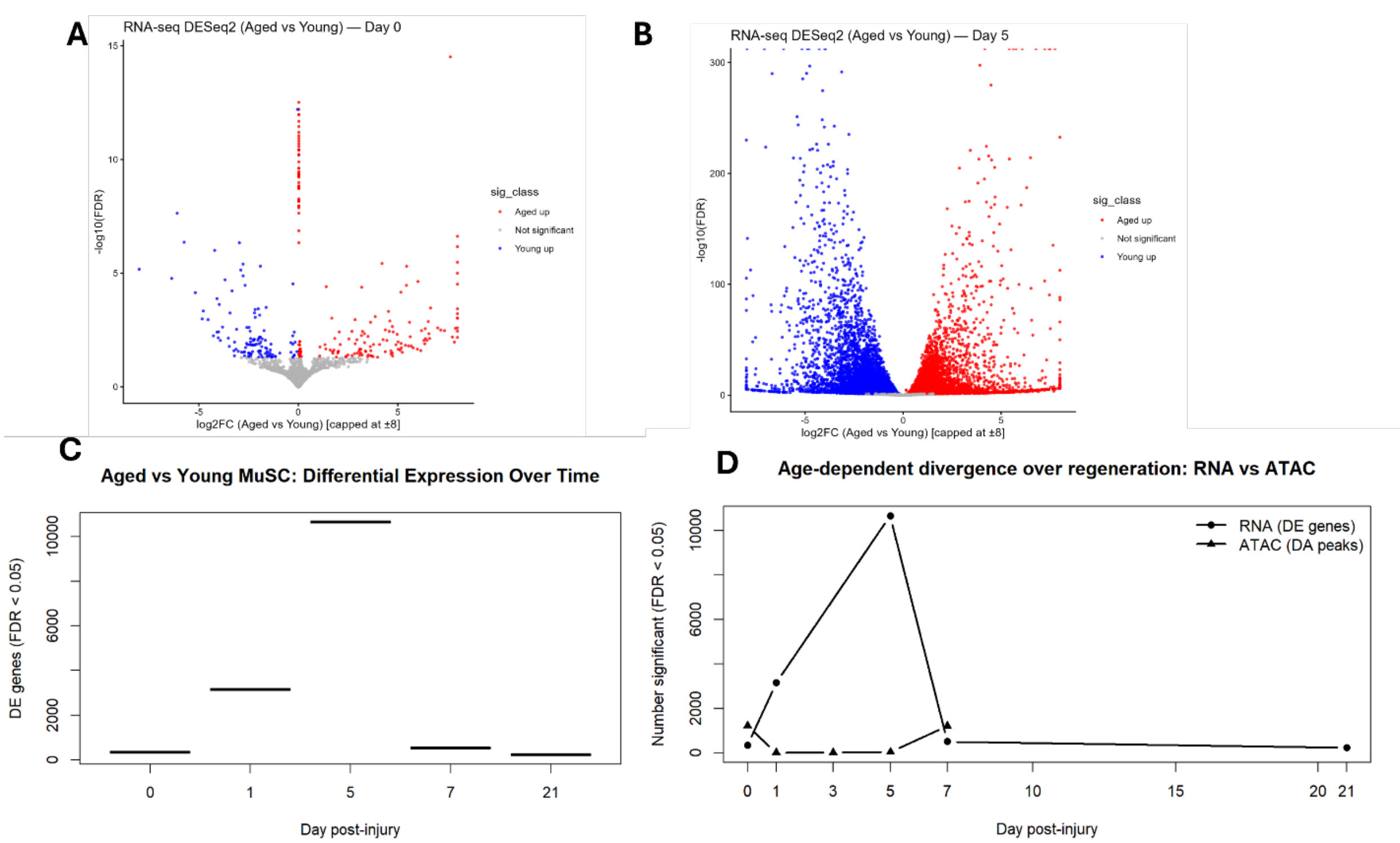

**Figure S3. Coordination between THR activity, nuclear receptor motif accessibility, and downstream transcriptional programs during muscle regeneration. (A)** Scatter plot showing the relationship between inferred THR activity (VIPER THR index) and aggregate nuclear receptor (NR) motif accessibility quantified by chromVAR from ATAC-seq data across matched biological replicates. Points represent individual samples from young (blue) and aged (red) mice. Solid lines indicate linear regression fits within each age group. **(B)** Temporal trajectories of NR DNA binding motif accessibility (chromVAR deviation z-scores) in muscle stem cells at 0,3 and 7 days post-injury. Points represent mean values ± SEM across biological replicates. (**C**) Relationship between THR motif accessibility and Foxo3 regulon activity at baseline (day 0), early regeneration (day 3), and late regeneration (day 7). Each point represents a biological replicate. Lines indicate linear regression within each age group. **(D)** Relationship between THR motif accessibility and Myod1 regulon activity across regeneration stages, plotted as in (C). Together, these analyses illustrate age- and stage-dependent differences in the coordination between chromatin accessibility at nuclear receptor-associated regions and downstream transcriptional programs during regeneration.

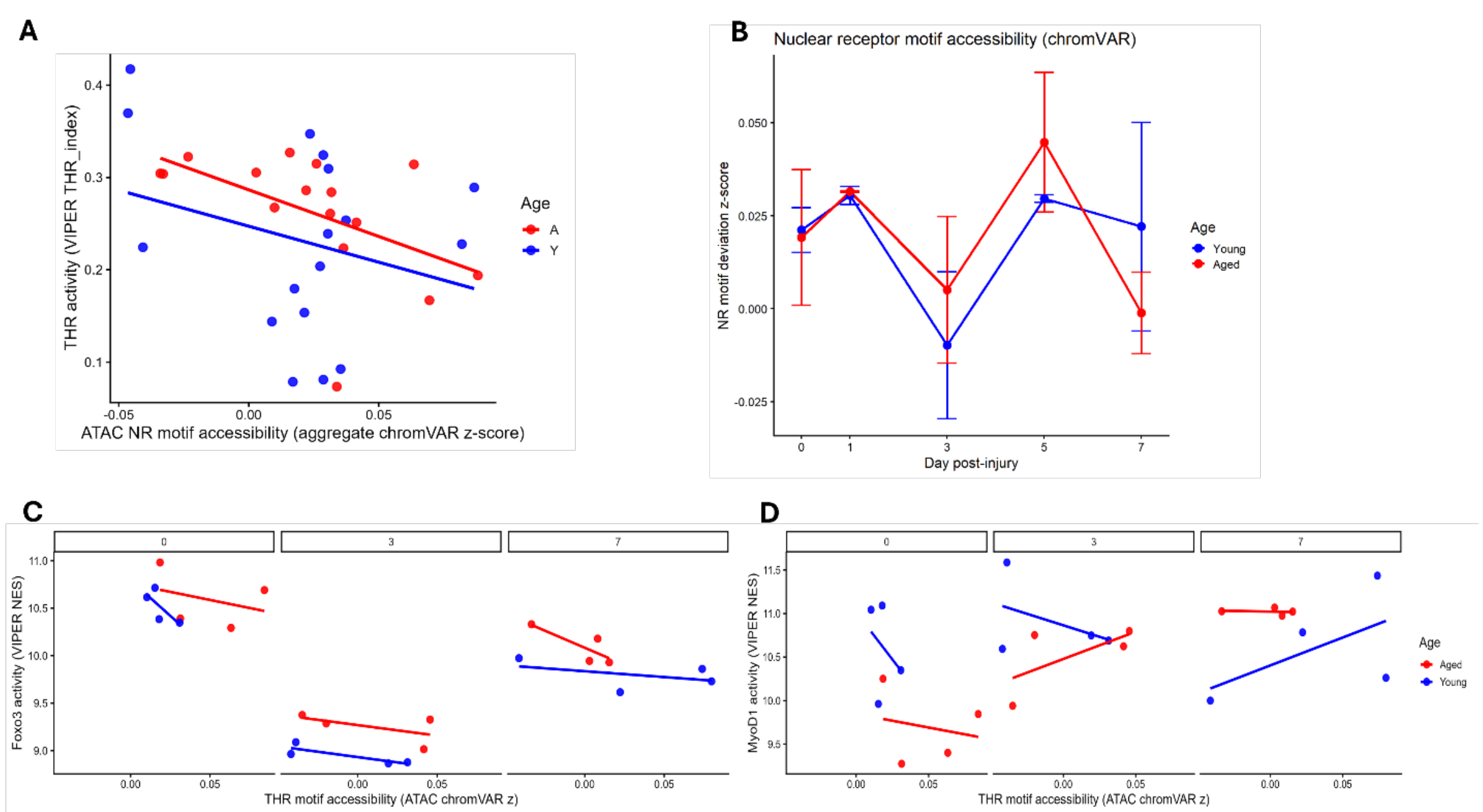

**Figure S4. Association between FAP thyroid hormone receptor activity and IL33 expression. (A)** Replicate-level relationship between FAP THRA activity and IL33 expression during regeneration. THRA activity was strongly associated with IL33 expression (Spearman ρ = 0.83, $p = 9.6 \times 10^{-5}$). **(B)** Replicate-level relationship between FAP THRB activity and IL33 expression (Spearman ρ = 0.70, p = 0.0036). These findings suggest that THR-dependent stromal programs may regulate immune-recruitment pathways linked to Treg accumulation and immune coordination during muscle regeneration.

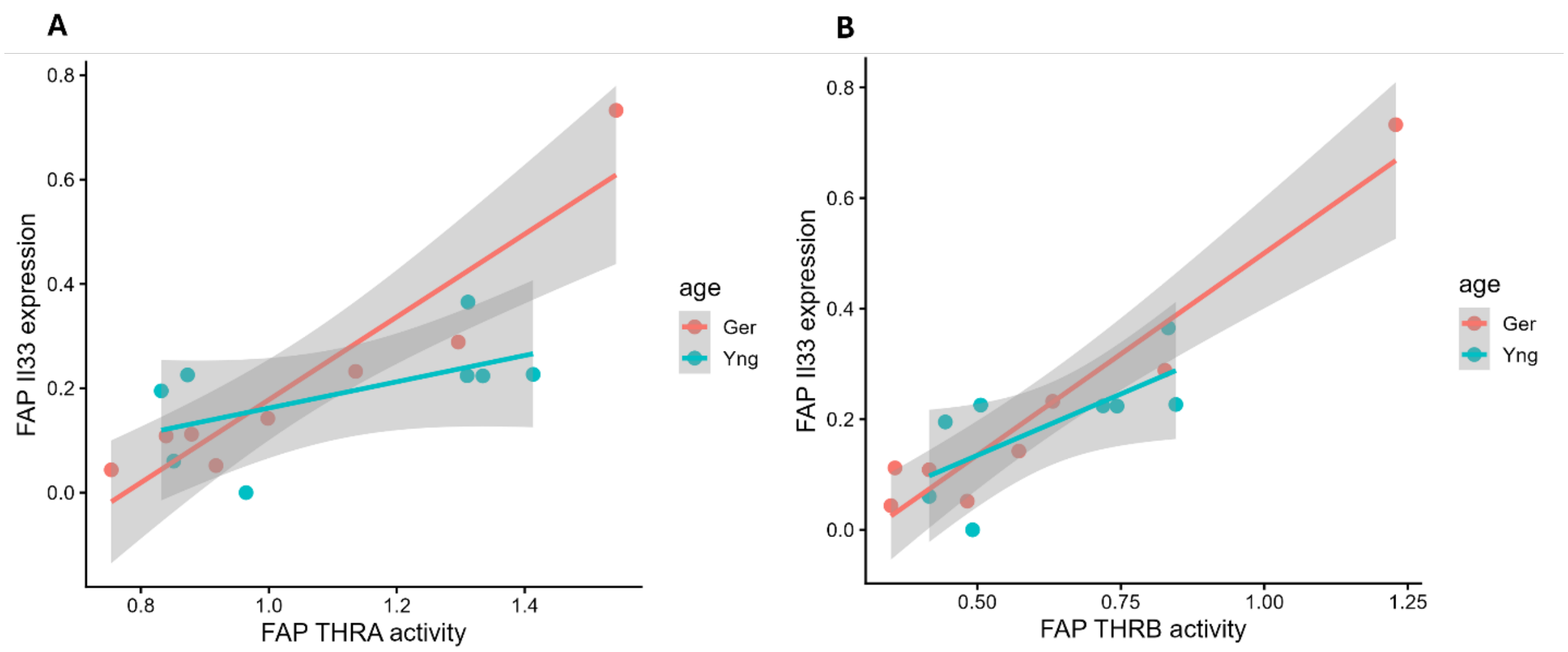


**Figure S5. Directional ligandreceptor (LR) pathway shifts in aging relative to young muscle during early regeneration.** Heatmaps display bootstrap-estimated differences in pathway activity (Δ = Aged - Young) for ECM/Integrin, IGF1, and Notch/Jagged ligand-receptor families at day 1 (D1) and day 3.5 (D3.5) post-injury, stratified by sender (rows) and receiver (columns). Directional pathway activity was computed using a hypothesis-guided ligand-receptor framework, in which interaction scores were defined as the product of mean ligand expression in sender populations and receptor expression in receiver populations, aggregated at the sample level. Differences between aged and young conditions were estimated using bootstrap resampling

across biological replicates (B = 300 iterations). Color scale indicates effect size, and black dots denote confidence intervals excluding zero. This analysis captures age-dependent remodeling of selected regenerative signaling pathways independent of THR activity**.**

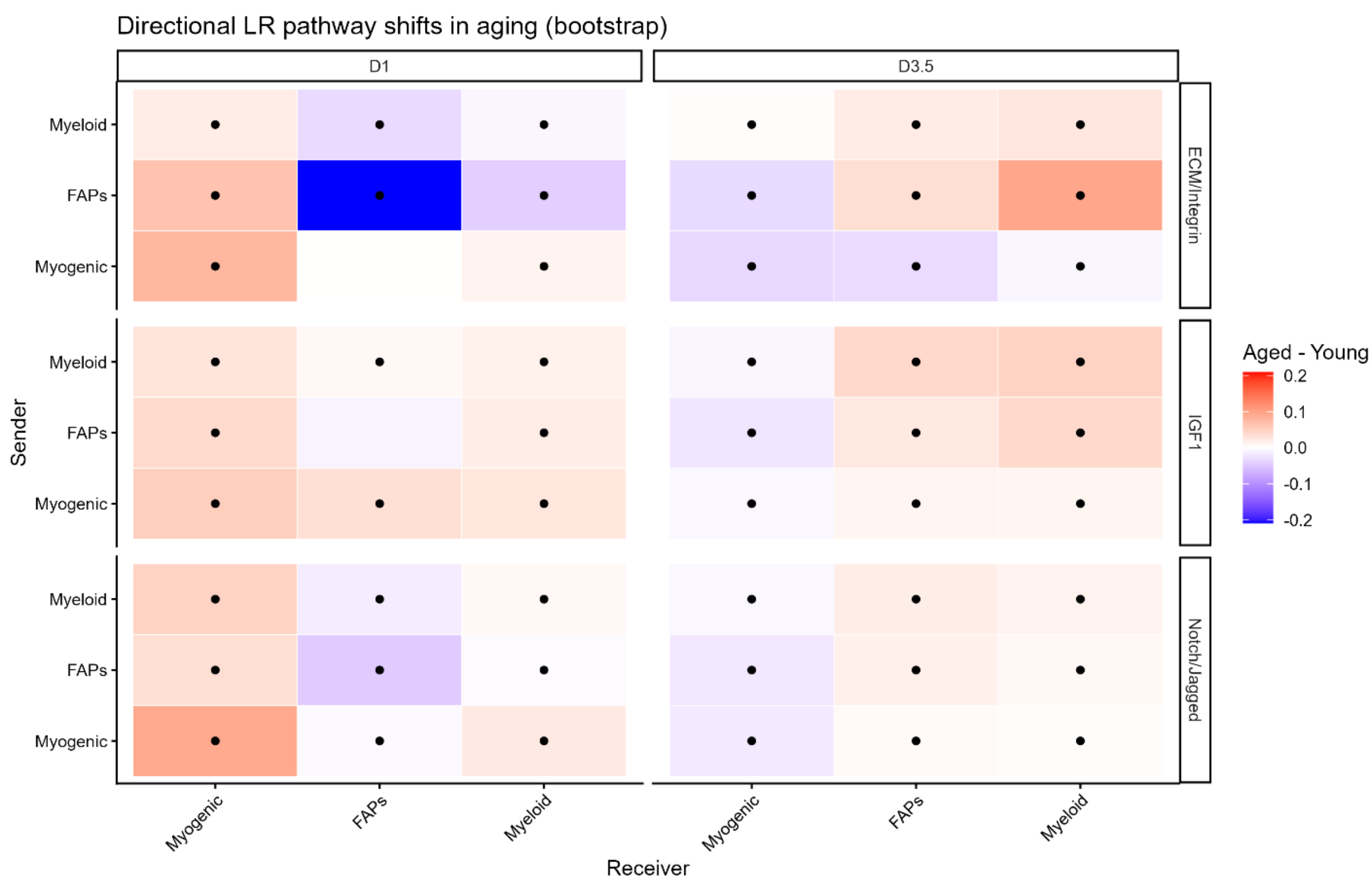


**Figure S6. Age-dependent rewiring of THR–program coupling across paracrine signaling contexts.** Heatmaps depict standardized regression coefficients (β) from linear models relating THR regulon activity to proliferative (Myc/E2F) and oxidative (Foxo/PGC1a) program scores as a function of ligand-receptor (LR) pathway context, lineage, regeneration stage, and age.

Models were fit separately for day 1 (D1) and day 3.5 (D3.5) post-injury in young and aged muscle. Rows indicate combinations of transcriptional programs and LR pathway families (ECM/Integrin, IGF1, Notch/Jagged), and columns indicate major muscle-resident lineages. Color intensity

represents the estimated THR-to-program coupling coefficient, with red indicating positive and blue indicating negative associations. This analysis evaluates how extracellular signaling context modulates THR-dependent transcriptional output, revealing age-dependent rewiring of THR-program coupling across paracrine environments.

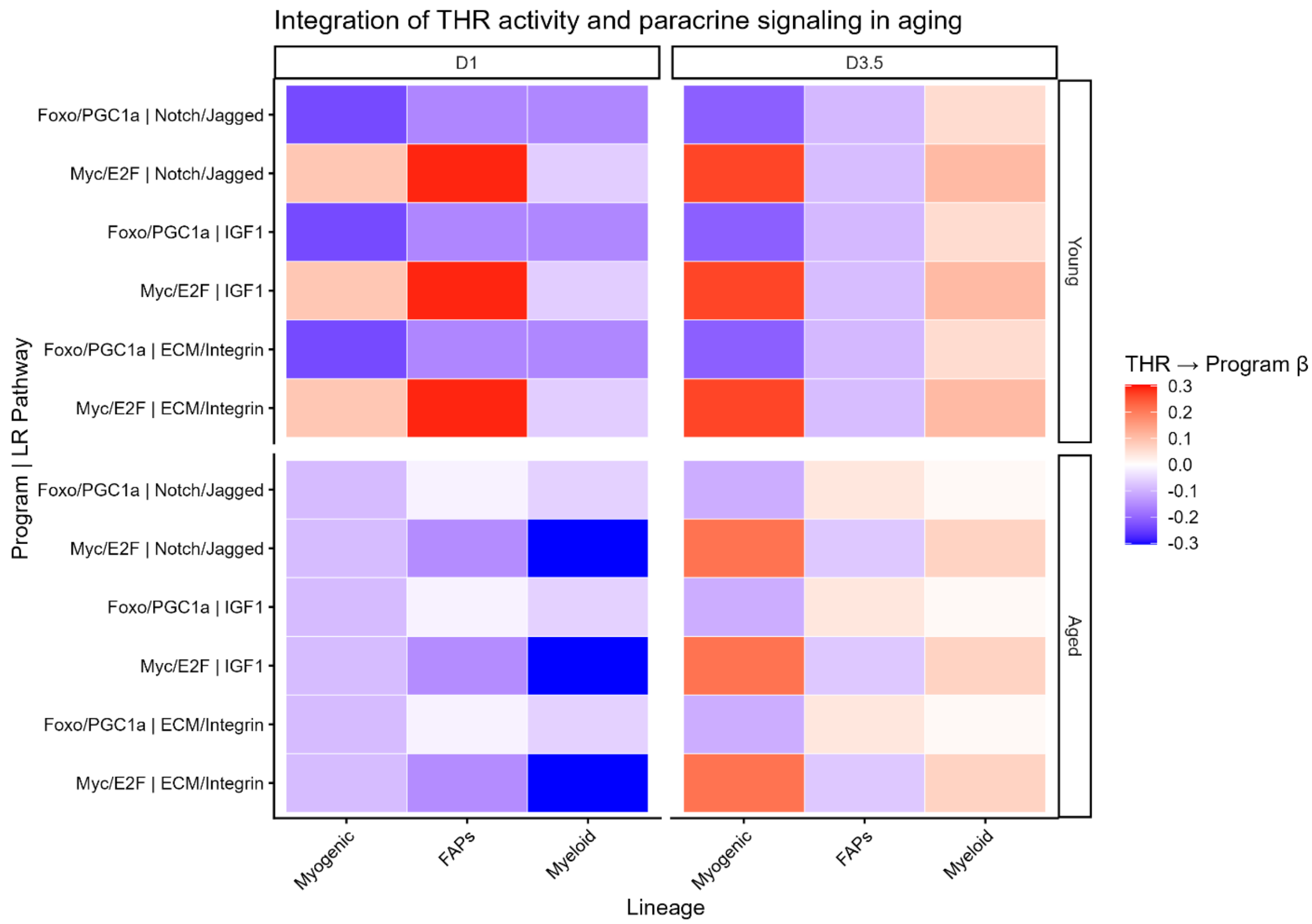

**Supplementary Table 1. Experimental and Literature Support for the Endocrine-Immune-Niche Integration Framework**

| Computational Finding | Supporting Experimental Evidence | References |
|---|---|---|
| **Section 3.1: THR-Regeneration Uncoupling** | | |
| Aging alters THR signaling dynamics without uniform suppression | Age-related tissue-level TH resistance with preserved but non-functional signaling; T4 replacement fails to improve aged muscle regeneration and induces atrophy; organ-specific uncoupling of TH availability from downstream responses | Mooradian 2019; Oliveira et al. 2025; Rakov et al. 2019 |
| Selective disruption of THR-MyoD coupling with preserved THR-Foxo3 | TRα loss-of-function downregulates MyoD1/Myf5; COUP-TFII interference selectively disrupts myogenic programs at TREs; downregulated TRα targets accumulate with aging (Col6a1); FoxO3/D2 pathway required for myogenesis, positioned upstream of MyoD | Sheng et al. 2024; Aguiari et al. 2021; Shi et al. 2025; Dentice et al. 2010 |
| Dio2/Dio3 machinery persists but becomes heterogeneous | D2/D3 constitutes cell-autonomous pre-receptor T3 control; D2 conversion doubles after injury; D3 protects proliferating satellite cells; reciprocal D2/D3 regulation under LSD-1/FoxO3 epigenetic control | Salvatore et al. 2014; Russo et al. 2021; Marsili et al. 2011; Dentice et al. 2014; Visser et al. 2016 |
| THR-associated chromatin remains accessible despite impaired output | Aging increases transcriptional heterogeneity via epigenetic drift at promoters without closing chromatin; aged HSCs show differentially open regions with altered functional output | Hernando-Herraez et al. 2019; Itokawa et al. 2022 |
| **Section 3.2: Stromal and Immune Coordination** | | |
| FAP-intrinsic THR-ECM regulatory axis | D2-produced T3 modulates FAP differentiation fate; COUP-TFII silencing reactivates ECM remodeling pathways in THRA-PV myoblasts | Luongo et al. 2025; Aguiari et al. 2021 |

| | | |
|---|---|---|
| THR-macrophage remodeling associations | THRA knockdown impairs proinflammatory macrophage polarization; hypothyroidism produces persistent proinflammatory macrophages with altered FAP/immune networks; D2 is require for macrophage inflammatory responce | van der Spek et al. 2018; Aguiari et al. 2026: Kwakkel et al. 2014 |
| THR-FAP-IL33 axis linking endocrine signaling to Treg recruitment | FAPs are the major IL-33-expressing cells in muscle; IL-33 administration to aged mice restores Treg accumulation and regeneration | Kuswanto et al. 2016 |
| Dual-mode architecture (direct myogenic + indirect paracrine) | scRNA-seq confirms T3 acts through both direct myogenic transcriptional control and indirect paracrine remodeling via FAP/immune networks | Aguiari et al. 2026 |
| **Section 3.3: Age-Dependent Niche Rewiring** | | |
| CM signaling strongest predictor of THR-high state; age-dependent attenuation | Aging extensively remodels the MuSC niche ECM; FAP-derived Smoc2 disrupts Integrin-β1/MAPK signaling, impairing MuSC function | Schüler et al. 2021 |
| Inflammatory pathway associations emerge with aging | Aged MuSCs undergo progressive enhancer rewiring that activates proinflammatory programs, with late-stage 3D genome reorganization reflecting loss of myogenic identity and heightened cytokine responsiveness | Shi et al. 2025 |
| Notch signaling negatively associated with THR activity in aging | Notch maintains satellite cell quiescence; aged Notch dysregulation impairs regenerative activation | Fujimaki et al. 2018; Peng at al. 2025 |
| **Section 3.4: Aging vs. Hypothyroidism** | | |
| Hypothyroidism: reduced THR activation, delayed program execution, and sustained | Hypothyroid mice show impaired regeneration with stalled MuSC cell cycle, reduced myogenic diversity, and persistent proinflammatory macrophages; $Spp1^{+}/Trem2^{+}$ signature | Aguiari et al. 2026; Hoeft et al. 2023 |

| | | |
|---|---|---|
| $Spp1^+/Trem2^+/Apoe^+$ macrophage activation | identified as conserved pro-fibrotic macrophage state across organs | |
| Aging: preserved activation but context-dependent execution | T4 replacement fails in aged muscle despite restored hormone levels; aged muscle exhibits local TH resistance distinct from systemic hypothyroidism | Oliveira et al. 2025; Mooradian 2019 |
| Distinct mechanistic modes converge on impaired regenerative output | Hypothyroidism and aging produce different regulatory signatures - signal deficit vs. integration failure - explaining why hormone replacement alone is insufficient in aging | Rakov et al. 2019; Oliveira et al. 2025 |

## Methods

**Data sources and experimental design.** We analyzed publicly available single-cell RNA-seq (scRNA-seq), bulk RNA-seq, and ATAC-seq datasets from regenerating skeletal muscle in young and aged mice (GSE143437, GSE232106, GSE121589)(1-4), as well as scRNA-seq data from hypothyroid and euthyroid mice (GSE311479)(5). GSE143437 comprises a time-course scRNA-seq dataset of murine skeletal muscle regeneration (days 0, 2, 5, and 7 post-injury). GSE232106 includes lineage-resolved scRNA-seq profiles from young and aged mice during early regeneration (days 1 and 3.5 post-injury). GSE121589 provides bulk RNA-seq and ATAC-seq data from muscle stem cells across multiple regeneration stages. Analyses focused on early regeneration time points to capture critical windows of satellite cell activation, proliferation, and differentiation. All datasets were processed using a consistent analytical framework to enable cross-dataset comparison of THR activity, transcriptional programs, and paracrine signaling. The hypothyroid dataset (GSE311479) was analyzed using the same scoring and modeling pipeline to facilitate direct comparison with aging-related perturbations. All computational analyses were performed in R (version 4.5.2) using Seurat(6), tidyverse, VIPER, CollecTRI, DESeq2 (with apeglm shrinkage), chromVAR, and lme4.

**Single-cell RNA-seq processing and cell type annotation.** Public scRNA-seq datasets (GSE232106 and GSE143437) were processed in Seurat and combined into a unified object for joint analysis. After quality-control filtering using dataset-appropriate thresholds, data were log-normalized, variable features were identified, and dimensional reduction was performed by principal component analysis. Neighbor graphs and UMAP embeddings were generated using the first 30 principal components, and graph-based clustering was performed using the Louvain algorithm at a resolution of 0.5. Cell identities were assigned using canonical lineage marker genes and manual curation. Lineage-resolved subsets were then used for downstream regulon, module-score, and pseudobulk analyses. For the hypothyroid dataset (GSE311479), previously

processed and annotated single-cell populations from our prior study were used, and downstream scoring and modeling were recomputed using the same analytical framework applied to the aging datasets. To support replicate-aware analyses, cells were grouped by biological sample within each age x lineage x regeneration-stage stratum. Normalized expression values were averaged across cells to generate lineage-resolved pseudobulk profiles. Cell-level regulon activity scores and transcriptional module scores were then summarized as sample-level means and used for downstream correlation and regression analyses.

**Bulk RNA-seq differential expression**. Bulk RNA-seq count matrices from GSE121589 were analyzed in R using DESeq2. Samples were annotated by age and regeneration stage, and samples lacking age information or not meeting predefined inclusion criteria were excluded prior to analysis. Differential expression between aged and young muscle was assessed separately at each time point using a design formula of ~ age, thereby isolating age-associated transcriptional differences within regeneration stage. Log2 fold-change estimates were shrunk using the apeglm (7) method to improve effect size stability. Statistical significance was assessed using Wald tests, and p values were adjusted for multiple testing using the Benjamini–Hochberg procedure.

**ATAC-seq processing and chromVAR analysis**. ATAC-seq data from GSE121589 were obtained as merged peak count matrices representing chromatin accessibility across muscle stem cell samples. Peaks were defined by genomic coordinates, and read counts per peak were used as input for downstream analysis. Sample metadata were curated and aligned to count matrices based on replicate identifiers. As in the RNA-seq analysis, a small subset of samples derived from Pax7-enriched populations was excluded to ensure comparability across the dominant myogenic population. For each regeneration stage, differential chromatin accessibility between aged and young samples was assessed independently using DESeq2. Peaks with low coverage (total counts ≤10 across samples) were excluded prior to analysis. Count data were modeled using a negative binomial framework with a design formula of ~ age, and statistical significance was

assessed using Wald tests with Benjamini-Hochberg correction. Peaks with FDR < 0.05 were considered differentially accessible. Transcription factor motif accessibility was quantified using chromVAR. Motif annotations were derived from curated transcription factor motif collections compatible with chromVAR, including motifs corresponding to JASPAR-defined transcription factor binding profiles. Motif-containing peaks were identified using position weight matrices, and chromVAR deviation z-scores were computed for each sample. Bias correction was performed using GC content- and accessibility-matched background peaks. Motif accessibility scores were analyzed at the sample level and compared across age groups and regeneration stages to assess temporal dynamics and variability of transcription factor-associated chromatin accessibility.

Associations between THR motif accessibility and THR regulon activity were evaluated using linear regression models at the sample level.

**Inference of thyroid hormone receptor activity and transcriptional execution.** THR activity was inferred using VIPER (Virtual Inference of Protein Activity by Enriched Regulon analysis) applied to curated *Thra* and *Thrb* regulons from the CollecTRI database. VIPER computes normalized enrichment scores (NES) that estimate transcription factor activity based on coordinated regulation of downstream target genes rather than receptor expression alone. A composite THR activity score (THR_mean) was calculated as the arithmetic mean of THRA and THRB regulon activity scores. The composition and overlap of THRA and THRB regulons were quantified to assess isoform specificity (Supplemental Methods). To mitigate the effects of single-cell sparsity and dropout, VIPER scores were computed at the single-cell level and subsequently aggregated at the biological replicate level within each lineage and regeneration stage. All downstream analyses were performed on replicate-level summaries rather than individual cells.

To quantify downstream transcriptional output of THR signaling, we used a consensome-based gene set derived from the Signaling Pathways Project (SPP)(8). Genes within the top 10% of the THR consensome (TC90) were defined as high-confidence targets, consistent with established

SPP conventions. The subset of TC90 genes present in the analyzed dataset was used to compute a transcriptional execution score (TRcons_1) as the average normalized expression across these genes. The full TC90 gene list and the subset used in this study are provided in 'SupportingDataValue' (THR consensome TC90 gene list). The execution score showed consistent positive association with inferred THR activity, supporting its validity as a proxy for downstream transcriptional output. Similar results were observed using alternative consensome thresholds, indicating robustness to the choice of cutoff.

To assess the relationship between THR activity and transcriptional execution, linear models were fitted at the single-cell level:

TRcons_1 ~ scale(THR_mean) * age * day + scale(nCount_RNA) + scale(percent.mt)

allowing separation of receptor activation from downstream transcriptional output and evaluation of age-dependent changes in their coupling. In regression models, β coefficients represent the direction and magnitude of association between variables (e.g., THR activity and transcriptional program scores) after accounting for covariates. These coefficients should be interpreted as conditional effect sizes rather than direct measures of coordination. In contrast, coordination between pathways was quantified using correlation-based metrics (e.g., Pearson or Spearman correlation of changes across biological replicates), which capture co-variation independent of model structure. Because variables were standardized prior to modeling, β coefficients are expressed in standardized units and can be compared across models, with larger absolute values indicating stronger associations.

**THR consensome-based transcriptional execution score.** To quantify downstream transcriptional output of THR signaling, a consensome-based execution score was computed using the Signaling Pathways Project (SPP) framework. The consensome approach integrates

differential expression evidence across multiple independent transcriptomic datasets to rank genes according to the strength and reproducibility of their regulatory association with a given signaling node. For thyroid hormone receptors, genes within the top 10% of the consensome ranking (TC90) were defined as high-confidence transcriptional targets. The THR consensome gene list was obtained from the SPP resource, and TC90 genes were intersected with the expressed gene set in each dataset. For each cell, a transcriptional execution score (TRcons_1) was calculated as the average normalized expression of these consensome-defined target genes. Expression values were derived from log-normalized scRNA-seq data. This score reflects the extent to which canonical THR-responsive transcriptional programs are executed, enabling separation of transcription factor activity (VIPER-derived) from downstream gene expression output.

To evaluate the relationship between THR activity and transcriptional execution, linear models were fitted at the single-cell level with TRcons_1 as the response variable and THR activity, age, and regeneration stage as explanatory variables. Interaction terms were included to assess age-dependent modulation of THR-execution coupling. Models were adjusted for sequencing depth and mitochondrial content using scaled covariates (nCount_RNA and percent.mt). This framework enabled quantification of the extent to which transcriptional output tracks with receptor activity and how this relationship is altered across aging and regeneration stages.

THR consensome TC90 gene list and THR_regulon_targets gene list are available in the file ‘SupportingDataValue”

**Quantification of TH-axis gene expression and T3 activation index.** Expression of thyroid hormone pathway components, including deiodinases (*Dio2*, *Dio3*) and transporters (*Slc16a2/MCT8*, *Slc16a10/MCT10*), was quantified from lineage-resolved pseudobulk expression profiles. For each gene, lineage, biological replicate, and age group, temporal changes during early regeneration were summarized as the difference between day 3.5 (D3.5) and day 1 (D1):

$\Delta$Expression = Expression_{D3.5} - Expression_{D1} (where expression values are log-normalized).

Expression values were derived from log-normalized pseudobulk expression profiles. To summarize transcript-level variation in thyroid hormone metabolism, a deiodinase balance index was computed at the sample level. For each lineage and biological replicate, mean expression of *Dio2* and *Dio3* was calculated from pseudobulk profiles, and the index was defined as: Deiodinase balance index = *Dio2 - Dio3*. This metric reflects the relative balance between activating (*Dio2*) and inactivating (*Dio3*) deiodinase expression programs and is interpreted as a transcript-level proxy rather than a direct measure of intracellular T3 concentration or enzymatic activity. Because deiodinase function is influenced by protein abundance, subcellular localization, post-translational regulation, and hormone transport, this index is used conservatively as a summary of TH-metabolism-related expression state.

Positive values indicate a bias toward local T3 activation, whereas negative values reflect increased thyroid hormone inactivation.

**Pathway activity scoring and coordination analysis.** Transcriptional program activity was quantified using regulon-based scores derived from CollecTRI-VIPER analysis. Functionally related regulons were grouped into biological modules representing key regenerative processes, including Myc/E2F-driven proliferation, Foxo/PGC1a-associated oxidative metabolism, extracellular matrix (ECM) remodeling, IGF signaling, and inflammatory response pathways. Module activity scores were computed as the mean normalized enrichment score (NES) across regulons within each functional group. To assess coordination between THR signaling and downstream regenerative programs, changes in module activity between early regeneration stages (D1 and D3.5 post-injury) were computed at the biological replicate level and correlated with corresponding changes in THR activity using Pearson correlation. A THR coordination index

was defined as the mean absolute correlation between changes in THR activity and changes in ECM and IGF module activity:

$$\text{Coordination Index} = (|r(\Delta THR, \Delta ECM)| + |r(\Delta THR, \Delta IGF)|) / 2$$

Robustness of coordination estimates was assessed using leave-one-out cross-validation (LOOCV), given limited replicate numbers ($n = 4$ per condition).

**Pathway enrichment of age-sensitive THRA interaction genes.** Sample-level mean FAP gene expression was calculated by averaging normalized single-cell expression across FAP cells within each biological replicate. For each gene, a linear model of the form

gene_expression ~ THRA_fap * age

was fitted, including both main effects and their interaction term. The p value associated with the interaction term was extracted and adjusted for multiple testing using the Benjamini-Hochberg procedure. Genes with adjusted p values $< 0.05$ were considered age-sensitive THRA interaction genes. Functional enrichment analysis was performed using g:Profiler (organism: *Mus musculus*), and significantly enriched biological process terms were summarized.

**Stromal proliferation score.** A FAP proliferation score was calculated to quantify stromal proliferative activity. The score was defined as the average normalized expression of canonical cell-cycle genes (Mki67, Pcna, Tk1, Top2a, Cenpf, Ube2c, and Birc5) across FAP cells within each biological replicate. This gene set captures classical markers of proliferation and DNA replication. Consistent with pathway enrichment results, this metric reflects a broader stromal proliferative competence state encompassing cell-cycle progression and genome-maintenance processes.

**Macrophage remodeling score.** A macrophage remodeling score was calculated to quantify remodeling-associated macrophage states during regeneration. The score was defined as the

average normalized expression of the macrophage remodeling genes *Apoe*, *Trem2*, and *Spp1* within macrophage cells for each biological replicate. Higher scores indicate increased adoption of a remodeling-associated macrophage transcriptional state.

**Detailed pathway activity and coordination analysis.** Transcriptional regulatory activity was inferred from scRNA-seq expression data using CollecTRI-VIPER, which estimates transcription factor and pathway activity based on curated regulatory networks. For each cell, normalized enrichment scores (NES) were computed for individual regulons and subsequently aggregated at the biological replicate level within each major lineage. To characterize higher-order biological programs, functionally related regulons were grouped into modules representing extracellular matrix remodeling, IGF signaling, inflammatory response, proliferative (Myc/E2F), and oxidative metabolism (Foxo/PGC1a) pathways based on curated biological annotations and CollecTRI pathway classifications. Module activity scores were calculated as the mean NES across regulons within each functional group. For each lineage and biological replicate, changes in module activity between day 1 (D1) and day 3.5 (D3.5) post-injury were computed. These changes were then correlated with corresponding changes in THR activity using Pearson's correlation coefficient. A coordination index was defined to quantify coupling between THR signaling and key regenerative pathways, focusing on ECM and IGF modules:

$$\text{Coordination Index} = (|r(\Delta\text{THR}, \Delta\text{ECM})| + |r(\Delta\text{THR}, \Delta\text{IGF})|) / 2$$

where r denotes the Pearson correlation coefficient across biological replicates. Absolute values were used to capture the strength of coupling independent of directionality.

To assess robustness given limited replicate numbers (n = 4 per condition), leave-one-out cross-validation (LOOCV) was performed, and variability in correlation estimates was reported. Correlation direction and sensitivity to individual replicates were explicitly evaluated.

**Curated ligand-receptor pathway analysis (hypothesis-guided).** Cell-cell communication was initially evaluated using a curated ligand-receptor framework focused on predefined signaling

pathways relevant to skeletal muscle regeneration. Canonical ligand–receptor pairs associated with extracellular matrix/integrin (ECM/Integrin), IGF1, and Notch/Jagged signaling were compiled from curated interaction resources and filtered based on expression in the scRNA-seq datasets. Interactions were filtered to retain only ligand–receptor pairs expressed in the scRNA-seq datasets. For each sender-receiver cell-type pair, directional interaction scores were computed as the product of mean ligand expression in sender populations and mean receptor expression in receiver populations, using pseudobulked expression values at the biological replicate level. Low-abundance interactions were excluded to reduce noise. To improve robustness and account for unequal cell numbers across samples, nonparametric bootstrap resampling (B = 300 iterations) was performed within lineage × age × time strata. For each bootstrap iteration, pathway-level scores were recomputed, and differences between conditions (Aged - Young) were recorded. Bootstrap distributions were summarized using mean estimates and percentile-based confidence intervals (2.5th–97.5th), and shifts were considered robust when confidence intervals excluded zero. Ligand-receptor pairs were grouped into predefined pathway families (ECM/Integrin, IGF1, Notch/Jagged), and pathway activity scores were calculated as the mean interaction strength across all receptor-mediated interactions within each family. These pathway-level scores were integrated with THR regulon activity and transcriptional program scores in mixed-effects and multivariable regression models to assess how predefined extracellular signaling contexts modulate THR-dependent transcriptional outputs during regeneration.

**Directional ligand-receptor family analysis.** Cell-cell communication was further quantified using a pseudobulk-based directional ligand-receptor interaction framework applied to scRNA-seq data. For each sample, cells were grouped by major cell populations (myogenic cells, FAPs, and macrophages), and mean normalized gene expression values were computed within each population to generate sample-level expression profiles. A curated ligand-receptor interaction set was constructed based on biologically plausible signaling molecules, including cytokines,

chemokines, extracellular matrix components, Notch ligands, and growth factors. To improve interpretability, ligand-receptor pairs were grouped into functional signaling families based on ligand identity, including inflammatory, extracellular matrix (ECM), Notch, and growth factor pathways.

Directional interaction scores were computed for each ligand-receptor pair as the product of mean ligand expression in the sender cell population and mean receptor expression in the receiver population, evaluated at the sample level for all combinations of sender and receiver cell types. Pathway-level interaction scores were calculated by averaging interaction strengths across all ligand-receptor pairs within each signaling family for a given sender-receiver pair.

These pathway-level scores were integrated with THR regulon activity using linear regression models to evaluate both (i) the relationship between THR activity and outgoing signaling (sender models) and (ii) the relationship between incoming signaling and THR activity (receiver models), with age and regeneration stage included as covariates. In earlier analyses, robustness of interaction estimates was also assessed using nonparametric bootstrap resampling (B = 300 iterations) within age x lineage x time strata. Bootstrap-derived confidence intervals were used to confirm stability of pathway-level differences, with consistent results observed between bootstrap-based and regression-based analyses.

**Integration of the two directional LR frameworks.** The two directional ligand-receptor analyses were designed to address complementary questions. The hypothesis-guided framework tested whether predefined signaling environments of particular biological interest modulate THR-dependent transcriptional programs. The broader directional LR family framework extended this analysis by comparing how multiple signaling families relate to THR activity across sender and receiver compartments. Together, these approaches enabled pathway-focused and systems-level characterization of endocrine-niche integration during muscle regeneration.

**Modeling THR-program coupling and paracrine context**. To assess how extracellular signaling modifies THR-dependent transcriptional programs, single-cell linear models were fitted with lineage-specific program scores (e.g., Myc/E2F or Foxo/PGC1a modules) as dependent variables:

ProgramScore ~ scale(THR_mean) * age * scale(LR_input) + day + CellType + scale(nCount_RNA) + scale(percent.mt)

where LR_input represents pathway-specific incoming ligand-receptor activity scores (e.g., ECM/Integrin, IGF1, Notch/Jagged). Three-way interaction terms (THR x age x LR_input) were used to test age-dependent modulation of THR–program coupling.

**Mixed-effects modeling of THR–paracrine interactions.** To account for replicate-level clustering and evaluate higher-order interactions, linear mixed-effects models were fitted using the lme4 package:

ProgramScore ~ scale(THR_mean) * scale(LR_input) * age * day + (1 | replicate)

Biological replicate was included as a random intercept to account for non-independence of cells derived from the same sample. The primary coefficient of interest was the four-way interaction term (THR × LR_input × age × day), which tests whether paracrine signaling reshapes THR-dependent transcriptional output across regeneration stages in an age-dependent manner. Models were fitted using restricted maximum likelihood (REML). Fixed-effect estimates (β), standard errors, and Wald statistics were extracted for inference. Model assumptions were assessed by inspection of residual distributions and fitted values.

**Generalized linear mixed-effects modeling of THR-high state probability.** To model predictors of entry into a THR-high transcriptional state, cells were classified into THR-high versus non-THR-high states based on predefined thresholds. A generalized linear mixed-effects model with a binomial link function was fitted:

THR_high ~ ECM_Input + IGF_Input + Notch_Input + age + day + scale(nCount_RNA) + scale(percent.mt) + (1 | replicate)

Regression coefficients are reported as log-odds with Wald z-statistics.

**Robustness and statistical considerations** Given the limited number of biological replicates (n = 4 per condition), robustness of correlation-based analyses was assessed using leave-one-out cross-validation (LOOCV). Correlation estimates were recomputed iteratively with one replicate removed, and the range of resulting values was reported to quantify sensitivity to individual samples. Multiple testing correction was performed using the Benjamini-Hochberg procedure (FDR < 0.05). Effect sizes and confidence intervals were reported alongside p values to emphasize biological relevance.

**Statistical analysis.** Correlation analyses were performed using biological replicates as independent units. Multiple testing correction was performed using the Benjamini-Hochberg method. Given limited sample sizes (n = 4 per condition), emphasis was placed on effect sizes and robustness metrics, including leave-one-out cross-validation (LOOCV) and bootstrap-derived confidence intervals. Multiple testing correction was performed using the Benjamini-Hochberg false discovery rate (FDR) procedure. Corrections were applied within each analysis family separately, including (i) gene-level differential expression analyses, (ii) gene-level interaction models (e.g., THRA x age), (iii) pathway enrichment tests, and (iv) ligand–receptor pathway comparisons. For correlation and regression analyses at the replicate level, emphasis was placed on effect sizes and consistency across datasets, with adjusted p values reported where applicable. For key replicate-level correlation analyses, robustness was assessed using leave-one-out cross-validation (LOOCV), in which the statistic of interest was recomputed after removing one biological replicate at a time. The range of resulting estimates was reported to quantify sensitivity to individual samples. Given limited biological replicate numbers, especially in the early-regeneration aging dataset, replicate-level correlations and higher-order interaction

models should be interpreted as estimates of directional association rather than precise effect-size measurements or definitive tests of mechanism.

### Software versions and computational environment

R version 4.5.2, Seurat v5.0, VIPER v1.44.0, DESeq2 v1.50.2, chromVAR v1.32.0, lme4 v1.1.38, tidyverse v2.0.0, ggplot2, OmnipathR 3.17.4. Platform: Windows 11 x64, CollecTRI regulons were accessed through decoupleR 2.16.0.

### Resource Table

| Resource | Source | Identifier |
|---|---|---|
| scRNA-seq young/aged muscle D1, D3.5 | Walter et al., 2024 | GEO: GSE232106 |
| scRNA-seq aged muscle D0,7 | Walter et al., 2024 | GEO: GSE232106 |
| scRNA-seq young D0,3,5,7 | De Micheli at al. 2020 | GSE143437 |
| Bulk RNA-seq + ATAC-seq MuSCs | Shcherbina  at al, 2020 | GEO: GSE121589 |
| scRNA-seq hypothyroid muscle | Aguiari et al., 2026 | GEO: GSE311479 |
| CollecTRI regulon database | Müller-Dott et al., 2023 | https://collectri.org |
| THR consensome gene list | Ochsner McKenna, 2020 | SPP Database |
| R statistical software | R core team | v4.5.2 |
| Seurat package | Hao et al., 2021 | CRAN |
| VIPER package | Alvarez et al., 2016 | Bioconductor |
| DESeq2 (with apeglm shrinkage) | Love et al., 2014; Zhu et al., 2018 | Bioconductor |

**Reference**


1. Walter LD, Orton JL, Hannah Fong EH, Maymi VI, Rudd BD, Elisseeff JH, et al. Single-cell transcriptomic analysis of skeletal muscle regeneration across mouse lifespan identifies altered stem cell states associated with senescence. *bioRxiv*. 2023.
2. Walter LD, Orton JL, Ntekas I, Fong EHH, Maymi VI, Rudd BD, et al. Transcriptomic analysis of skeletal muscle regeneration across mouse lifespan identifies altered stem cell states. *Nat Aging*. 2024;4(12):1862-81.
3. De Micheli AJ, Laurilliard EJ, Heinke CL, Ravichandran H, Fraczek P, Soueid-Baumgarten S, et al. Single-Cell Analysis of the Muscle Stem Cell Hierarchy Identifies Heterotypic Communication Signals Involved in Skeletal Muscle Regeneration. *Cell Rep*. 2020;30(10):3583-95 e5.
4. Shcherbina A, Larouche J, Fraczek P, Yang BA, Brown LA, Markworth JF, et al. Dissecting Murine Muscle Stem Cell Aging through Regeneration Using Integrative Genomic Analysis. *Cell Rep*. 2020;32(4):107964.
5. Aguiari P, Villani V, Liu YY, Carraro G, Brent GA, Perin L, et al. Hypothyroidism impairs skeletal muscle regeneration after injury by altering myogenic and nonmyogenic pathways. *JCI Insight*. 2026;11(6).
6. Hao Y, Hao S, Andersen-Nissen E, Mauck WM, 3rd, Zheng S, Butler A, et al. Integrated analysis of multimodal single-cell data. *Cell*. 2021;184(13):3573-87 e29.
7. Zhu A, Ibrahim JG, and Love MI. Heavy-tailed prior distributions for sequence count data: removing the noise and preserving large differences. *Bioinformatics*. 2019;35(12):2084-92.
8. Ochsner SA, and McKenna NJ. No Dataset Left Behind: Mechanistic Insights into Thyroid Receptor Signaling Through Transcriptomic Consensome Meta-Analysis. *Thyroid*. 2020;30(4):621-39.
